# Uranium contamination mediating soil and ore microbial community assembly at four mining sites, South China

**DOI:** 10.3389/fmicb.2025.1553072

**Published:** 2025-02-19

**Authors:** Hongyu Chen, Yizhi Sheng, Shuaidi Wang, Yu Chen, Zhiyuan Qiao, Huaming Guo, Hailiang Dong

**Affiliations:** ^1^Center for Geomicrobiology and Biogeochemistry Research, State Key Laboratory of Biogeology and Environmental Geology, China University of Geosciences, Beijing, China; ^2^Frontiers Science Center for Deep-time Digital Earth, China University of Geosciences, Beijing, China; ^3^School of Environment, Tsinghua University, Beijing, China; ^4^MOE Key Laboratory of Groundwater Circulation and Evolution & School of Water Resources and Environment, China University of Geosciences, Beijing, China

**Keywords:** uranium mining, microbial community, high-throughput sequencing, community assembly processes, radioresistance

## Abstract

Uranium mining presents significant environmental challenges, particularly through radiological contamination affecting soil and water bodies. While soil microbial communities are known to be influenced by geochemical factors like pH and nutrient availability, their responses to severe uranium contamination in mine tailing environments remain poorly understood. This study investigated microbial community distributions in soils and uranium ores at four uranium mining sites in South China to explore microbial adaptations to uranium contamination. Uranium concentrations ranged from 170 to 18,000 mg/kg, with the most severely contaminated samples dominated by Cyanobacteria, which comprised up to 49.17% of the microbial community. Proteobacteria, such as *Sphingomonas*, were also abundant, indicating their roles in radiation resistance, while Acidobacteriota and Actinobacteria showed negative responses to uranium. Addition of lime to neutralize the acidity in mine tailings led to an increase in Gemmatimonadaceae, a family commonly found under oligotrophic conditions. Multivariate statistical analyses confirmed uranium concentration as the primary factor influencing microbial composition, along with pH values, total nitrogen, and contents of Fe_2_O_3_ and SiO_2_ in soils. Co-occurrence network analysis suggested that extremely high uranium concentrations disrupted microbial interrelationships, reflecting communities lived more independently and adopted strategies to cope with the intense selective pressure. Intriguingly, dispersal limitation governed 90% of community assembly in high-uranium environments (>10,000 mg/kg), suggesting more isolated ecological niches. Deterministic processes such as heterogeneous and homogeneous selection only dominated the community assembly at relatively moderate to low uranium levels. These findings provide insights into the ecological dynamics of uranium-contaminated sites and related bioremediation strategies.

## 1 Introduction

Uranium (U) is a critical resource and plays a central role in nuclear energy generation, defense industries, and radiation-based medical treatments (Belkin, 2024). However, the environmental consequences of uranium mining are profound and challenging (Agboola et al., 2020; Carvalho, 2017; Haneklaus, 2021). Mining activities contribute to radioactive uranium contamination, particularly in soils and aquatic systems (Matshusa and Makgae, 2017). Such contamination adversely impacts surrounding ecosystems, including soils, water bodies, and local biota, necessitating effective solutions to mitigate these environmental risks (Boteva et al., 2016; Campbell et al., 2015). The persistence of radioactive contamination further complicates remediation efforts (Campbell et al., 2015), creating long-term challenges that destabilize ecosystems and threaten the health of microbial communities.

In uranium-contaminated environments, microorganisms play a pivotal role in uranium mobility and transformation through various biochemical pathways. In organic matter-rich sediments, elevated organic carbon levels enhance microbial activity, promoting uranium immobilization via reductive processes, which subsequently affect its solubility and migration behavior (Chen et al., 2022; Cumberland et al., 2016; Li R. et al., 2024; Xia et al., 2022; Zhang et al., 2021). Dissimilatory metal-reducing bacteria (DMRB), such as *Geobacter* and *Shewanella*, utilize uranium as an electron acceptor during anaerobic respiration (Fredrickson et al., 2002; Holmes et al., 2002). In this process, electrons are transferred from electron donors to uranium via membrane-bound enzymes, such as cytochromes like OmcA and MtrC in *Shewanella* (Dong et al., 2023a,b; Shi et al., 2016; Sheng et al., 2021a). The soluble U(VI), typically in the form of uranyl ions (UO_2_^2+^), is transformed into uraninite (UO_2_) (Lovley and Phillips, 1992; Rogiers et al., 2022), a less toxic and less mobile solid. This reduction decreases the mobility of uranium in the environment, effectively sequestering it into a more stable form (Dong et al., 2022; Dong and Lu, 2012; Lovley and Phillips, 1992; Zhang et al., 2011).

In aquatic environments this process generally occurs at low uranium concentration (e.g., <4–5 mg/kg), and microbial reduction is more effective; however, elevated uranium concentrations (e.g., 6–10 mg/kg) may inhibit microbial activity, reducing the efficiency of uranium immobilization (Lakaniemi et al., 2019; Lian et al., 2024). In contrast, sediment microbial communities exhibited a stronger tolerance to uranium contamination, achieving 96.5% removal of 7.14 mg/kg uranium from water by reduction and absorption (Zeng et al., 2020). Furthermore, Mondani et al. (2011) identified microbial activity of rod-like shape microorganisms in soils at uranium concentration of 255,000 mg/kg. Similarly, Xia et al. (2020) isolated *Microbacterium* sp. 6–1 from 20,680 mg/kg U bearing rock samples in South China. Specific microbial groups, such as *Holophaga*, *Sphingobacterium* and *Rhudobium*, have been identified in uranium-contaminated sediments with uranium concentrations of approximately 100 to 4,000 mg/kg (Mumtaz et al., 2018; Sutcliffe et al., 2017). Despite progress in isolating uranium-resistant pure cultures, microbial community structures and their responses to severe uranium contamination remains poorly understood (Babich et al., 2021; Sanyal et al., 2024).

The mobilization of uranium in soils at the mine trailing sites is highly complicated (Yin et al., 2019). During acid leaching, acidic solutions (e.g., sulfuric acid) are injected into uranium deposits to extract uranium, generating uranium-bearing tailings that are typically deposited in tailing dams. These tailings create a highly acidic environment due to residual acid and ongoing geochemical reactions. To mitigate the acidity, lime (calcium oxide, CaO) is often applied to neutralize the acidic tailings (Csovari et al., 2004; Opitz et al., 1984; Robertson et al., 2019), a treatment that could significantly alter the indigenous microbial communities. Such alterations in microbial communities, in turn, can influence the biogeochemical behavior of uranium, impacting its mobility within the tailing dams. However, the distribution of microbial communities in tailing dams under neutralization treatments remains uncharacterized.

The primary objective of this study is to examine the microbial community structure and diversity across different sampling sites in uranium mining regions of South China, focusing on how microorganisms adapt to and respond to uranium contamination, associated geochemical conditions, and environmental treatments. The uranium mining regions of South China are characterized by unique geological and environmental features, especially across the mining belts intersecting Guangdong, Jiangxi, and Hunan provinces. These areas were chosen for the study due to their significance in China’s uranium mining history (Chi et al., 2020; Hu et al., 2017; Zhao and Liu, 2024). Prolonged mining activities have resulted in significant radiological contamination and ecological risks (Li H. et al., 2024; Shi et al., 2021; Yuan et al., 2024). This study aims to uncover the ecological adaptation mechanisms of microorganisms in extreme environments, identify potential microbial resources for uranium remediation, and provide a scientific basis for sustainable strategies to mitigate environmental impacts of uranium mining.

## 2 Materials and methods

### 2.1 Site description and sample collection

**Figure 1:**
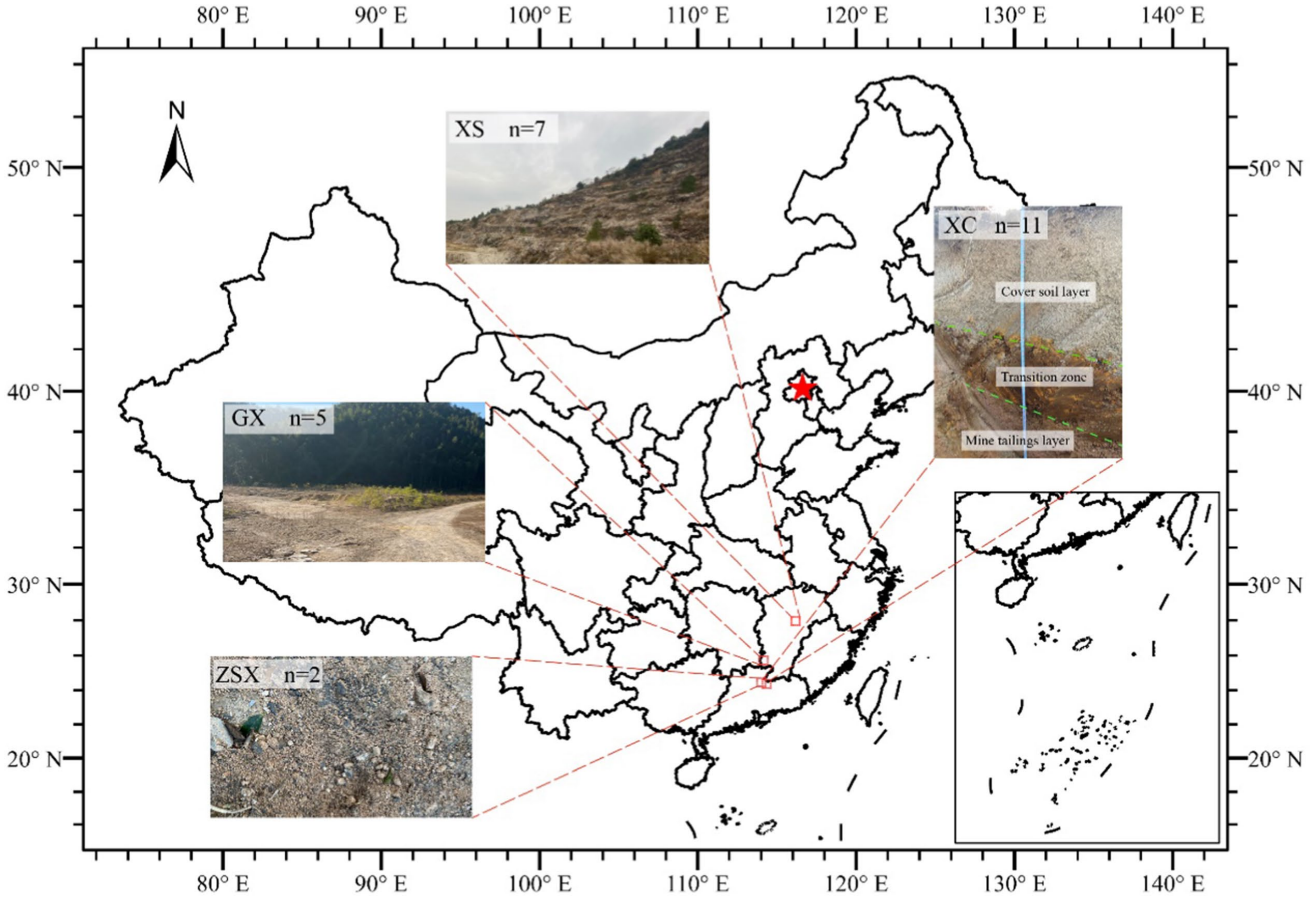
Geographic distribution of sampling sites in South China. XS: Xiangshan, Fuzhou, Jiangxi province (7 rock weathering samples). XC: Xiaoca, Shaoguan, Guangdong province (11UMT samples from vertical profile of UMT dam). GX: Guxuan, Ganzhou, Jiangxi province (5 UMT samples from GX dam). ZSX: Zhushanxia, Shaoguan (2 samples from open mining pit). The map was adapted from (Jiang et al., 2023).

The South China uranium deposits constitute the largest source of uranium in China (Luo et al., 2017). Most of these deposits are granite-hosted uranium ores, contributing approximately 30% of the country’s uranium production over the past three decades (Luo et al., 2017; Wang et al., 2017). The sampling sites include XiangShan (XS), ZhuShanXia (ZSX), GuXuan (GX), and XiaoCa (XC), with uranium mining tailings (UMT) covered in most areas (Figure 1). A FD-3013 environmental gamma radiation monitor (Xian Yima Optoelec Co., Ltd.) was employed for *in situ* measurements of radiation in soil and ore samples. Radiation readings, recorded in nC/(kg·h) or μSv/h, were converted to mg/kg following the instrument’s specifications.

The XS site is an open mineral pit. A total of 7 ore weathering surface samples were collected, which exhibited significantly high radiation dose (25.19–167.93 μSv/h, 2,700–18,000 mg/kg) compared to surrounding rocks (0.43 μSv/h). These sampling sites were characterized by their yellowish surfaces and the presence of minor faults and joints, indicative of enhanced weathering and structural alteration.

The ZSX site is another open mineral pit. Two soil samples were collected near the ore rocks. The first sample (ZSX-1-1) was collected at a uranium ore placement site, where the radiation exposure dose measured at the center of the ore was approximately 1750.53 mg/kg U. The second one (ZSX-1-2) was located one meter away from ZSX-1-1, with an exposure dose of 43.86 mg/kg U. Notably, no other nearby soil samples exhibited similarly high radiation levels, making these sampling points distinctive for their elevated radiation exposure doses.

The GX site is a UMT dam, with the sampling site located at its top. A total of 5 samples were collected at one-meter lateral intervals. To ensure the integrity of the samples, approximately 1 cm of the surface layer, including any vegetation, was removed prior to sampling. The uranium concentration of this site ranged from 900 to 16,000 mg/kg.

The XC site, a UMT dam, ceased mining and refining activities after its decommissioning in 2012 (Liu et al., 2024). Samples were collected directly from the dam using sterilized excavator buckets to obtain a vertical profile extending to a depth of 210 cm. The excavator buckets were sterilized with ethanol and flame and then air-cooled before use. The dam profile comprised three distinct layers: the cover soil layer (0–100 cm), the transition zone (100–150 cm), and the mine/mill tailing layer (150–210 cm). The transition zone was treated with calcium oxide (CaO) to neutralize the low pH caused by uranium acid leaching. The mine tailing layer comprised coarse mill tailings of a grain size of 6–10 mm and a brownish color. A total of 12 samples were collected at depths of 0, 15, 30, 50, 70, 90, 110, 130, 150, 170, 190, and 210 cm. Due to plant root residues, the surface sample at 0 cm was excluded from microbial community analysis.

All samples were collected in December 2020 using sterilized tools to ensure contamination-free handling. Moss and fallen leaves, if present, were carefully removed from the sampling sites. Samples were collected in individually sterilized, packaged sampling cups. After collection, the samples were labeled and immediately stored in dry ice containers (Yongjie Co., Ltd.) to preserve their integrity for subsequent geochemical and microbial analyses.

### 2.2 Geochemical characterizations

In the laboratory, moisture content was determined using the gravimetric method (Woodward and Gadd, 2019). Samples were dried to a constant weight in a convection oven, and then ground with an agate mortar and pestle to a particle size of less than 2 mm (Whyte and Greer, 2005). For pH measurement, a 1:5 (w/v) mixture of soil/rock and 1 mol/L KCl was prepared, shaken for 60 min on a mechanical shaker to create a homogeneous suspension, and then measured using a pH meter (Thermo Scientific Orion Star A211).

Major element contents of soils, including SiO_2_, TiO_2_, Al_2_O_3_, Fe_2_O_3_, MnO, MgO, CaO, Na_2_O, K_2_O, and P_2_O_5_ were analyzed using a Shimadzu 1800 X-ray fluorescence spectrometer (XRF) on fused glass disks at the State Key Laboratory of Geological Process and Mineral Resources (GPMR), China University of Geosciences, Beijing. The analytical precision was within ±3% precision for elements with concentrations >1.0 wt.% and ± 10 for elements <1.0 wt.%.

Total carbon (TC) and total nitrogen (TN) contents were determined using an Elementar Vario Macro Cube element analyzer, with 200 mg of sample per measurement. The analytical uncertainty for both TC and TN contents was less than 0.5%. For total organic carbon (TOC) analysis, approximately 500 mg of powdered sample was treated with 0.1 N HCl to remove inorganic carbon, followed by drying at 60°C. A 150 mg aliquot of treated sample was then analyzed using an Analytik-jena multi N/C 2100 s series analyzer equipped with a nondispersive infrared (NDIR) sensor. The combustion furnace temperature was maintained at 900°C for precise TOC determination.

### 2.3 DNA extraction, PCR amplification, and sequencing

DNA extraction from UMT samples is particularly challenging due to their high heavy metal contents and low microbial biomass. To address this, our laboratory has developed a specialized extraction protocol for efficient nucleic acid recovery from such environments (Guo et al., 2021). For each sample, approximately 35 g of each sample, stored at −80°C, was thawed and used for DNA extraction.

The quality of the extracted nucleic acids was assessed using a NanoDrop 1,000 spectrophotometer (Thermo Fisher Scientific, Waltham, MA, United States), ensuring the A260/A230 and A260/A280 ratios over 1.80 for all samples. The concentration of DNA was determined using a Qubit fluorometer and Qubit dsDNA HS Assay Kit (Invitrogen).

The quality-controlled DNA samples were amplified targeting the 16S rRNA V4 region using the bacterial and archaeal universal primers 515F (5′- GTGYCAGCMGCCGCGGTAA-3′) and 806R (5′- GGACTACHVGGGTWTCTAAT-3′). Each primer was tagged with a unique 12-bp barcode at the 5′ end to distinguish different samples. Detailed descriptions of the primers and barcoding strategy were described in our previous study (Hou et al., 2013). Amplifications were performed in triplicate using the Platinum SuperFi II Green PCR Premix (Invitrogen, Thermo Fisher Scientific) in a 25 μL reaction volume. The PCR conditions included an annealing temperature of 60°C, an extension time of 10 s, and a total of 30 cycles.

PCR products were verified by electrophoresis on a 1% TAE agarose gel at an electric field strength of 4 V/cm for 30 min, using DL2,000 (Takara) as a DNA marker. The agarose gel was prepared with a 10,000x SYBR Safe DNA Gel Stain (Thermo Fisher Scientific). After electrophoresis, DNA bands were visualized using a Tanon 1,600 Gel Imaging System (Tanon Shanghai, China). The target bands around 300 bp were excised, and DNA was purified using a Gel Extraction Kit (Omega Bio-Tek, Cat. No. D2500). The three replicates for each sample were pooled and eluted in a total volume of 50 μL sterile, DNase-free water.

The concentration of the gel-purified products was determined using Qubit, and samples were pooled in equal DNA mass for each sample to construct a library. The amplicon libraries were prepared following the standard protocol of the NEBNext^®^ Ultra™ II DNA Library Prep Kit for Illumina^®^ (New England Biolabs, United States). The constructed libraries were sequenced on the Illumina NovaSeq 6,000 platform with paired-end 250 bp reads (PE250) at Guangdong Magigene Biotechnology Co., Ltd. (Guangzhou, China).

### 2.4 Sequence processing and statistical analysis

The 16S rRNA V4 region amplicon sequencing data were processed using QIIME2 (version 2023.2) (Bolyen et al., 2019). Quality control and denoising were performed using the DADA2 (Callahan et al., 2016) plugin to obtain amplicon sequence variants (ASVs).

For taxonomic classification, the SILVA 138.1 (Quast et al., 2013) database was used. The “ssu_nr99” version of the SILVA 138.1 reference files was first filtered to extract only the 16S rRNA V4 region corresponding to the primers 515F and 806R (described former). A naive Bayes classifier (Bokulich et al., 2018) was then trained on this extracted region to ensure accurate taxonomic assignment of ASVs.

After taxonomic classification, taxa identified as mitochondria, chloroplasts, or those classified only to the domain level as “Bacteria;” or “Archaea;” were filtered out from the ASV table using the taxa filter-table function in QIIME2.

For downstream analyses, the ASV table was rarefied to 19,285 reads per sample (the minimum ASV reads among all samples) using the feature-table rarefy (Weiss et al., 2017) function in QIIME2 to standardize sequencing depth across all samples. Rarefaction curves suggested sufficient sequencing depths after standardization (Supplementary Figure S1). This rarefaction step helps to minimize biases caused by uneven sequencing depth. The rarefied ASV table was then utilized for subsequent analyses, including diversity assessment, community composition analysis, and various statistical evaluations. The raw sequence data has been deposited in the Genome Sequence Archive database (BioProject: PRJNA1200928).

After processing and quality control of the amplicon data, alpha and beta diversities were calculated using the phyloseq package (version 1.48.0) (McMurdie and Holmes, 2013) in R (version 4.4.1). For beta diversity analysis, a distance matrix was calculated using the Bray-Curtis distance, followed by principal coordinate analysis (PCoA) to visualize differences in microbial community composition. To evaluate the Pearson’s correlation among physicochemical factors, the Hmisc package (version 5.1–3) in R was used, and the results were visualized using the ggcorrplot package (version 0.1.4.1).

To explore the relationships between microbial communities and environmental factors, we employed multivariate data analysis methods including canonical correspondence analysis (CCA), redundancy analysis (RDA), and the Mantel test. Given that the length gradient of the ASV table was greater than 3.0, CCA was chosen for subsequent analyses (Borcard et al., 2011; Lepš and Šmilauer, 2003). Prior to CCA, to reduce errors due to collinearity among environmental factors, we used the vegan package (version 2.6–6.1) to assess the variance inflation factor (VIF) of environmental variables, considering a VIF greater than 20 to indicate severe collinearity issues. The packfor (version 0.0–8) R package was employed to evaluate the significance and cumulative contribution of each factor. The significance threshold was set at 0.05, and the cumulative contribution threshold was set to the adjusted explained variance of all factors in the CCA. Upon VIF analysis, the final environment factors selected for CCA analyses were Fe_2_O_3_, pH, TC, U concentration, and MnO.

Mantel test was performed to evaluate the relationship between microbial community and each environmental parameter at different taxonomic levels using the “mantel” module version 2.2.1 in Python (version 3.9.7) (Mantel, 1967). The distance matrix for each environmental parameter was computed using Euclidean distance, while Bray-Curtis distance was used for microbial communities at class, order, family, and genus levels. After identifying taxa that were significantly correlated with U concentration (Pearson, *p*-value <0.05), taxa tables were aggregated at different taxonomic levels and visualized using Chiplot. Correlations between environmental factors and microbial communities were calculated using Kendall’s tau, with weighted clustering based on Euclidean distance without normalization.

Network analyses of microbial communities were performed using R (v4.4.0) and Gephi (v0.10). To explore the relationships between ASVs and environmental factors, correlation networks were first constructed between physicochemical parameters and microbial taxa at the ASV level. Co-occurrence networks were then built to analyze the relationships among ASVs. ASVs were filtered based on a threshold of 0.0005, and Spearman correlation coefficients were calculated using the Hmisc package. Correlations with an absolute value greater than 0.5 and *p*-values less than 0.05 were retained. A weighted undirected network was constructed using the igraph package within R, and isolated nodes were removed. The final network was exported in GraphML format for visualization in Gephi.

To infer community assembly processes, a null model approach was used to calculate the beta nearest taxon index (βNTI), as previously described (Ning et al., 2020; Stegen et al., 2013; Stegen et al., 2012). When βNTI values are greater than +2, the observed community differences are significantly higher than expected by chance, indicating that deterministic selection is the dominant force in community assembly, specifically heterogeneous selection driving significant differences between communities. When βNTI values are less than −2, it suggests that community differences are significantly lower than expected by chance, indicating another homogeneous selection, where similar environmental pressures act on different samples. When βNTI values fall between −2 and + 2, the observed differences do not significantly deviate from random expectations, suggesting that stochastic processes, such as ecological drift and dispersal limitation.

## 3 Results and discussion

### 3.1 Soil physicochemical parameters

**Figure 2:**
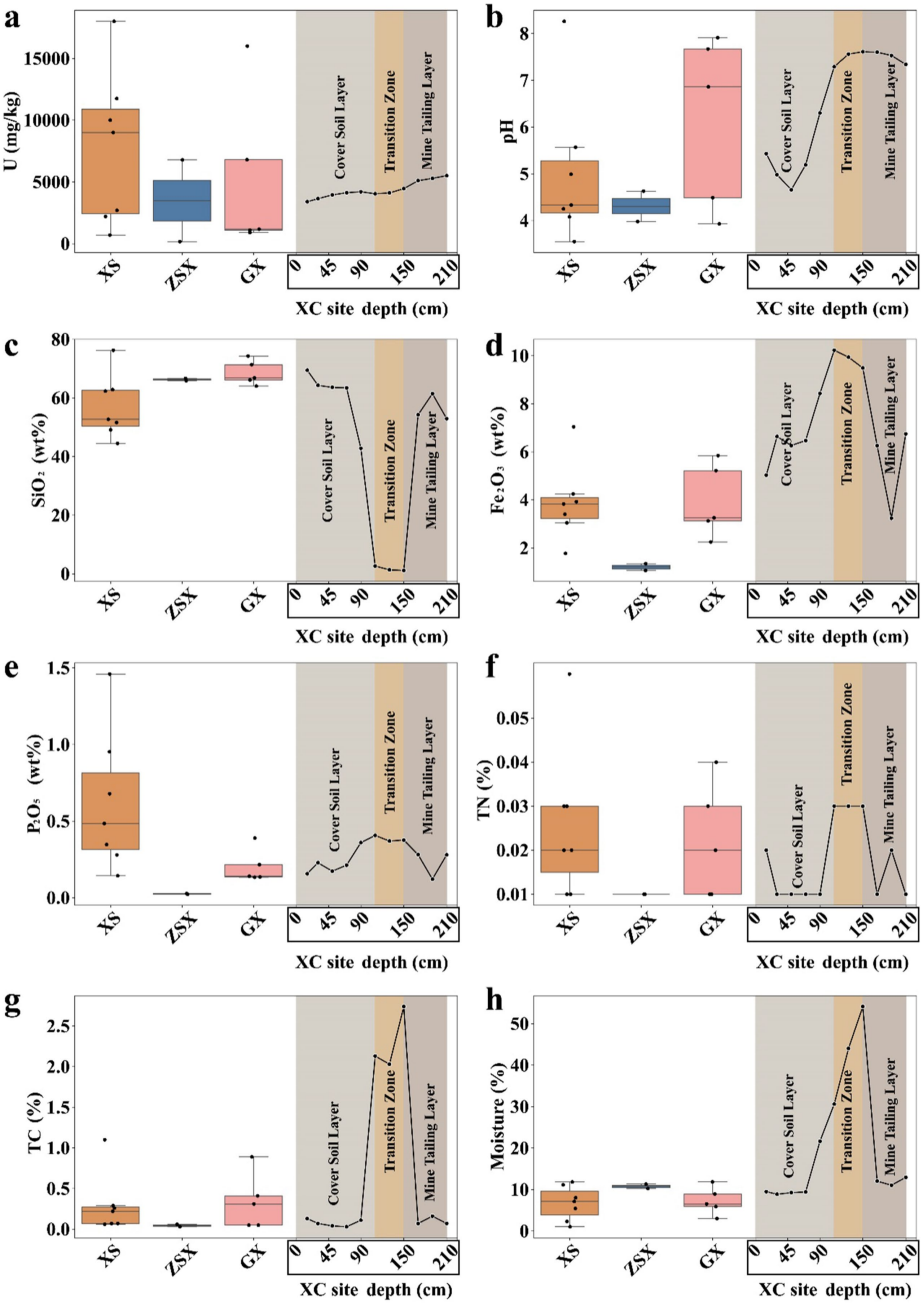
Physicochemical parameters of samples across different sites. **(A)** Uranium (U) concentration, **(B)** pH, **(C)** SiO₂ content, **(D)** Fe₂O₃ content, and **(E)** P₂O₅ content, **(F)** TN content, **(G)** TC content, and **(H)** moisture. Box plots of physicochemical parameters included samples at XS, ZSX, and GX sites, while the line chart included samples at XC site from different vertical layers. At site XC, the depth intervals were as follows: 0–110 cm corresponded to the Cover Soil Layer, 110–150 cm to the Transition Zone, and 150–210 cm to the Mine Tailing Layer.

Significant variations in physicochemical properties of soil and ore samples were observed across different uranium mining areas (Figure 2). The highest U concentration of 18,000 mg/kg was recorded in XS area (average 7,764 mg/kg, Figure 2A). Other sites exhibited comparable levels of U, with site-specific variations: ZSX (3477.5 mg/kg), GX (5195.0 mg/kg), and XC (4347.9 mg/kg). Notably, a sample with an exceptionally high U concentration of 16,000 mg/kg sample was also identified in GX. In the vertical profile of XC mine, U concentrations increased with depth, ranging from 3,399–4,126 mg/kg in the cover soil layer to 5,099–5,510 mg/kg in the mine tailing layer (*t*-test, *p* < 0.05), indicating a prolonged history of uranium deposition in the deeper layers. The uranium concentrations observed in this study were notably higher than those reported in previous studies. For instance, the Ranger uranium mine (RUM) in Australia showed uranium concentrations ranging from 0 to 4,000 mg/kg in sediment mesocosms (Sutcliffe et al., 2017). Similarly, Mumtaz et al. (2018) classified U concentrations >900 mg/kg as a “very high” category in the RUM Land Application Areas. In contrast, past studies have shown that the downstream samples of XC dam site exhibited U concentration ranging from 47.5–123.3 mg/kg (Yuan et al., 2024) and 28.1–70.1 mg/kg (Liu et al., 2024). Other studies within the same research area showed U concentration of 20.2–43.5 mg/kg (Zhang et al., 2023). However, these previous studies in the XC mine did not examine the samples collected in close proximity to the uranium open-pit mining areas.

The pH levels across most sampling points were mostly acidic (3.55 to 4.63), apart from slightly alkaline conditions observed in a few samples at GX and the deeper layers of XC (~7.5) (Figure 2B). The acidic soil conditions are likely influenced by acid rain, which is common in South China, where Latosols, known for their inherent acidity, are widespread (Kuang et al., 2008; Qin et al., 2012; Xu et al., 2003). Previous simulation experiments have demonstrated that soils leached by acid rain often displayed a negative correlation between pH and K, Mg, and Ca (Zhang et al., 2007). As pH decreases, the leaching of these cations from the soil increases. However, our samples did not show such correlations (Supplementary Figure S2). Instead, the observed acidity was more likely a result of the rock weathering and the effects of mining activities (Sheng et al., 2016). At the XC site, the application of CaO elevated the pH in the deeper layers to 7.29–7.61, creating a slightly alkaline environment in the transition layer. Moreover, the infiltration of pore water from the transition zone into the underlying mine tailing layer likely contributed to its neutral to alkaline pH conditions. The elevated pH reduced the mobility of uranyl ions by decreasing their solubility under alkaline conditions, thereby limiting their migration and promoting uranium accumulation in the deep layers of the XC site (Ragnarsdottir and Charlet, 2000) (Figure 2A).

SiO₂ concentrations varied significantly across regions, ranging from 42.80 to 76.24% in XS, ZSX, and GX samples (Figure 2C). These values aligned with those typically observed in early weathered granites or rhyolites (Nesbitt and Young, 1982). Among all the sites, the GX samples exhibited the highest degree of weathering. Previous studies have demonstrated that rock weathering can influence microbial community structures by altering nutrient availability and modifying the physical properties of the rocks (Chen et al., 2023; Choe et al., 2020). Notably, the SiO₂ content in the XC transition zone was significantly lower (1.37 to 2.66%) compared to other layers (52.92 to 69.43%) (*t*-test, *p* < 0.001). This was a direct result of CaO additions, reducing the relative proportion of rock-derived SiO_2_.

The Fe₂O₃ concentration also showed regional variations. The GX and XS samples showed similar Fe₂O₃ concentrations, averaging 5.03 and 4.72%, respectively (Figure 2D). In contrast, the ZSX samples displayed a notably lower Fe₂O₃ content, averaging around 3.40%. The XC sites displayed higher Fe₂O₃ levels, with the transition zone reaching the highest concentration at 9.78%. This elevated Fe₂O₃ content reflected the intentional addition of CaO, which likely contained Fe_2_O_3_, to mitigate acidic leachates and subsequently altering the geochemical composition of this layer.

Similar to the U distribution, P_2_O_5_ contents in the XS samples exhibited the highest average values, ranging from 0.14 to 1.46 wt.% (Figure 2E). In contrast, the ZSX samples exhibited the lowest P_2_O_5_ concentration, averaging approximately 0.02 wt.%, followed by GX. At the XC site, the transition zone showed significantly higher concentrations (0.37 to 0.41 wt.%) compared to the other two layers. Again, this enrichment may result from the co-deposition of phosphorus with lime or other remediation materials used at the site. Nonetheless, a positive correlation was observed between P₂O₅ and U concentrations across the study sites (Supplementary Figure S2, Pearson’s r = 0.47, *t*-test *p* < 0.05). Similar findings have been shown in the Ranger uranium mine with P₂O₅ concentrations of 0.13 to 0.15 wt.% in uranium-contaminated sediments (Sutcliffe et al., 2017). Positive correlation between uranium and phosphorus can be explained by a isomorphous substitution mechanism, where uranium substitutes for calcium in apatite (a phosphate mineral containing 42.06% P₂O₅) (Clarke and Altschuler, 1958; Luo et al., 2009; Pan and Fleet, 2002). When calcium, uranium, and phosphate reach certain levels, microorganisms such as *Caulobacter crescentus* can form extracellular precipitates, mitigating uranium toxicity by preventing its intracellular accumulation (Hu et al., 2005).

TN content was uniformly low across all samples, ranging from 0.01 to 0.06 wt.% (Figure 2F; Supplementary Table S1), which may constrain microbial activity in these environments (Sheng et al., 2023; Zhou et al., 2024). The distribution of TC and moisture content was also similar across the sampling sites, with TC values below 0.5 wt.% (Figure 2G) and moisture content below 10% (Figure 2H), except for elevated levels observed in the XC transition zone (~2 wt.% for both). Moreover, the TOC in the XC transition zone was at least an order of magnitude higher than in other zones (Supplementary Table S1).

### 3.2 Microbial community structures across different sites

From the initial 3,249,771 V4 sequences, 2,367,282 sequences remained after quality control and chimera removal (Supplementary Table S2). These sequences yielded a total of 20,834 ASVs, spanning 58 phyla and 1,301 genera. The microbial richness, as measured by Chao1 index, ranged from 314 to 3,449, and microbial diversity, as measured by Shannon index ranged from 3.74 to 7.39, reflecting overall moderate to high richness and diversity. Inverse Simpson index (InvSimpson), ranging from 9.40 to 789.39, revealed variations in the degrees of dominance among communities (Supplementary Table S3). Among all the sampling sites, the GX samples displayed the highest alpha-diversity, followed by ZSX. The transition layer at the XC site under the influence of lime treatment exhibited the lowest alpha-diversity, consistent with its unique physical–chemical properties (Figure 2). Other groups did not show significant variations when grouped by sampling location or uranium level (Supplementary Figure S3).

**Figure 3:**
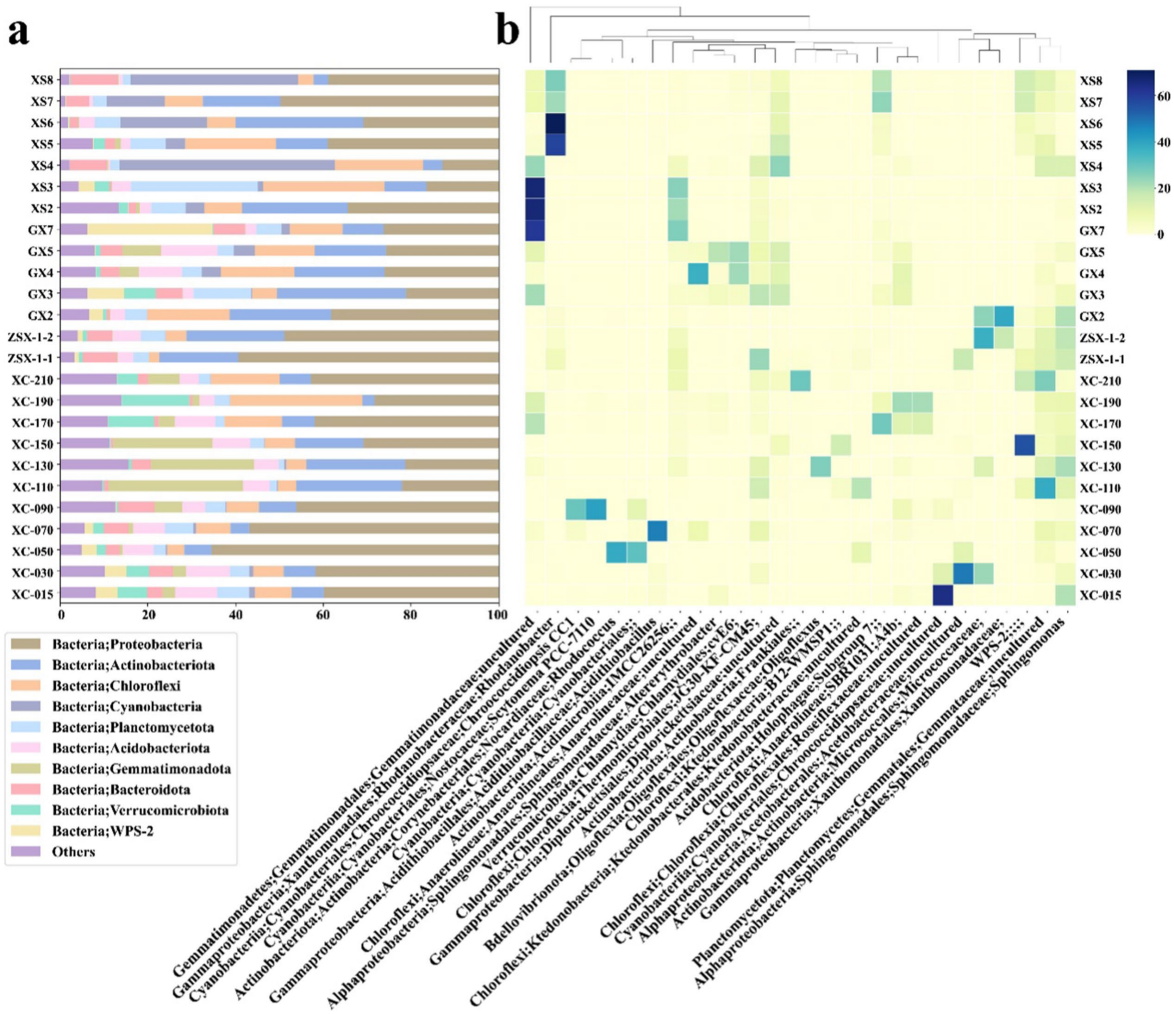
Microbial community composition of samples from the study area. **(A)** The relative abundance of microbial phyla, with the top 10 most abundant phyla represented. The category “Others” included unassigned taxa and phyla with lower abundances. **(B)** The relative abundance at the genus level, highlighting the top two genera from each sample to ensure a representative selection, resulting in a total of 27 genera.

The microbial communities of the two XS samples, XS4 and XS8 with U concentrations of 18,000 mg/kg and 11,750 mg/kg, respectively, were dominated by Cyanobacteria, accounting for approximately 49 and 38% of the total microbial communities, respectively (Figure 3A; Supplementary Table S4). The predominant genera within Cyanobacteria were *Chroococcidiopsis* and *Scytonema*, both known for their resilience in extreme environments, such as those with high ultraviolet radiation and oxidative stress, conditions commonly associated with surface exposures (Dong et al., 2007; Pathak et al., 2022; Rastogi et al., 2014). Their abundance suggested their crucial roles in mitigating oxidative stress induced by uranium exposure (Chao et al., 2023; Yang et al., 2024). Proteobacteria was another dominant phylum, with its relative abundance at ~40% and ~ 15% in XS8 and XS4, respectively. Within this group, *Sphingomonas* was particularly prevalent (~16% in XS8, Figure 3B), known for its metal resistance and biodegradation capabilities (Nilgiriwala et al., 2008). Actinobacteria had lower relative abundance than other samples (4.40% in XS4 and 3.37% in XS8, Figure 3A), suggesting that extreme uranium concentrations may suppress their growth or activity, consistent with a previous study (Suzuki and Banfield, 2004). Similarly, Acidobacteria exhibited minimal presence in these samples (0.51% in XS4 and 0.86% in XS8, Figure 3A), likely indicating its sensitivity to high uranium concentrations and acidic pH at these sites (Figure 2B) apparently did not promote their growth (Figure 2B). Gemmatimonadota were also scarce (0.36% in XS4 and 0.24% in XS8).

Both GX4 and GX5 had high uranium concentrations (16,000 mg/kg and 6,800 mg/kg, Figure 2A) and relatively high pH levels (~7) and SiO_2_ content. Different from the XS samples, microbial communities in these samples were more diverse, with no single dominant species. Proteobacteria was still prominent (~20–40%), and Acidobacteria maintained a significant presence (9.72% in GX4 and 12.92% in GX5), demonstrating their adaptability to uranium and circumneutral pH levels, again inconsistent with the traditional classification of Acidobacteria as acidophiles. A previous study showed that some members (such as Blastocatellia and *Bryobacter*, both thrived in GX4 and GX5) of Acidobacteria can adapt to a wide range of pH conditions (Kielak et al., 2016). Gemmatimonadota were also present in notable amounts (4.53% in GX4 and 8.77% in GX5, Figure 3A), which possess the capability for anoxygenic photosynthesis and are typically present in oligotrophic environments (Mujakic et al., 2022). Samples GX2, GX3 and GX7 had relatively lower uranium concentrations (900–1,175 mg/kg) but were also characterized by a dominance of Proteobacteria, particularly the *Sphingomonas* (6.28 to 7.02%, Figure 3B), and an uncultured genus from Acetobacteraceae (1.56 to 9.10%). In GX3, taxa at the genus levels affiliated to Frankiales and Gaiellales accounted for 9.10 and 5.55%, respectively. In GX7, the *Candidatus* Eremiobacterota (also known as WPS-2) dominated, representing 27.71% of the microbial community (Figure 3B). Frankiales, Gaiellales and *Candidatus* Eremiovacterota have been previously reported to be associated with selenium-contaminated environments (Rosenfeld Carla et al., 2018), extreme deep-sea habitats (Chen et al., 2021), and acidic environments in polymetallic deposits (Gavrilov et al., 2019). Unlike the XS site, where photosynthesis was primarily catalyzed by Cyanobacteria, microbes capable of anoxygenic photosynthesis constituted a large proportion of the microbial communities in the GX samples (Gemmatimonadota in high-U samples and WPS-2 in low-U samples). This result suggested that anoxygenic photosynthesis may play a significant role in these oligotrophic environments, potentially providing essential carbon sources (Ward et al., 2019).

The ZSX samples, ZSX-1-1 (high uranium, 6,785 mg/kg) and ZSX-1-2 (low uranium, 170 mg/kg), exhibited similar microbial community structures despite their difference in uranium concentrations, suggesting that other factors may govern microbial composition. Both samples were predominated by Proteobacteria (50–60%), specifically the Xanthomonadaceae (unclassified at genus level) and *Sphingomonas*. Actinobacteria, represented by Micrococcaceae (unclassified at genus level), were also present but showed a decrease in relative abundance as uranium concentrations rose (Figure 3), consistent with the findings at the XS sites.

At the XC site, the microbial community distribution was probed along soil depth. In the cover soil layer (15–100 cm), Proteobacteria was dominant, with the genus *Rhodanobacter* being particularly abundant (6.99–37.20%, Figure 3B). *Rhodanobacter* is known for its association with heavy metal resistance and uranium detoxification under well-oxygenated conditions (Green et al., 2012). In the transition zone (100–150 cm) with high a pH (Figure 2B), Gemmatimonadaceae (unclassified at genus level) became dominant (22.63 to 30.66%, Figure 3A). Acidobacteria also had a relative abundance between 5.57 and 9.28%. In the mine tailing layer (150–210 cm), *Chlamydiales bacterium* cvE60 (3.88–9.70%) and Anaerolineaceae (unclassified at genus level, 4.57–23.86%) were predominant. The presence of Chlamydiales with versatile metabolic capabilities in extreme environments, such as anoxic deep-sea sediments, suggested their potential to survive in uranium-rich tailings (Collingro et al., 2020; Dharamshi et al., 2020). Anaerolineaceae, known for their adaptation to anaerobic conditions, also showed an adaptation to uranium exposure (Zeng et al., 2019).

To sum up, Proteobacteria consistently dominated the microbial communities in uranium-contaminated environments. *Sphingomonas* and *Rhodanobacter* demonstrated adaptability to uranium stress, likely due to their heavy metal resistance mechanisms and metabolic versatility. Cyanobacteria were primarily found in surface samples with high uranium concentrations and sufficient light availability, underscoring their high tolerance to these extreme environments (Dong et al., 2007). Their oxygenic nature may enable mechanisms for mitigating oxidative stress under extreme uranium conditions. Additionally, the extracellular polymeric substances (EPS) and organic ligand production may bind and immobilize uranium (Guo et al., 2025). Acidobacteria demonstrated an unexpectedly broad pH tolerance (from 3.55 to 8.26), remaining abundant even in neutral to alkaline soils. In fact, Acidobacteria showed a positive correlation with pH (*t*-test, *p* = 0.04, Supplementary Table S5) but a negative correlation with contents of Al_2_O_3_, K_2_O and P_2_O_5_ (*t*-test, *p* < 0.05, Supplementary Table S5). Actinobacteria were sensitive to extremely high uranium concentrations. However, in samples with moderate uranium levels, their abundance either remained stable or even increased, suggesting a threshold effect where uranium concentrations above 10,000 mg/kg negatively impacted their viability. Gemmatimonadota thrived in samples with higher pH levels, such as the XC’s transition zone under neutralization treatment, indicating that alkalinity played a crucial role in their proliferation.

### 3.3 Multivariate analysis of microbial communities and physicochemical properties

To identify key factors influencing microbial community composition across different sampling locations, multivariate statistical analyses were conducted. Five physicochemical parameters—pH, Fe₂O₃, U concentration, MnO and TC—were selected based on the VIF results that were used to remove collinearity among environmental factors. The data points in CCA plot are color-coded based on the uranium concentration levels. U concentrations were classified into three categories using the gap-weighting method (Thiele, 1993): low (170–3,643 mg/kg), average (3,950–5,288 mg/kg), and high (5,510–18,000 mg/kg).

**Figure 4:**
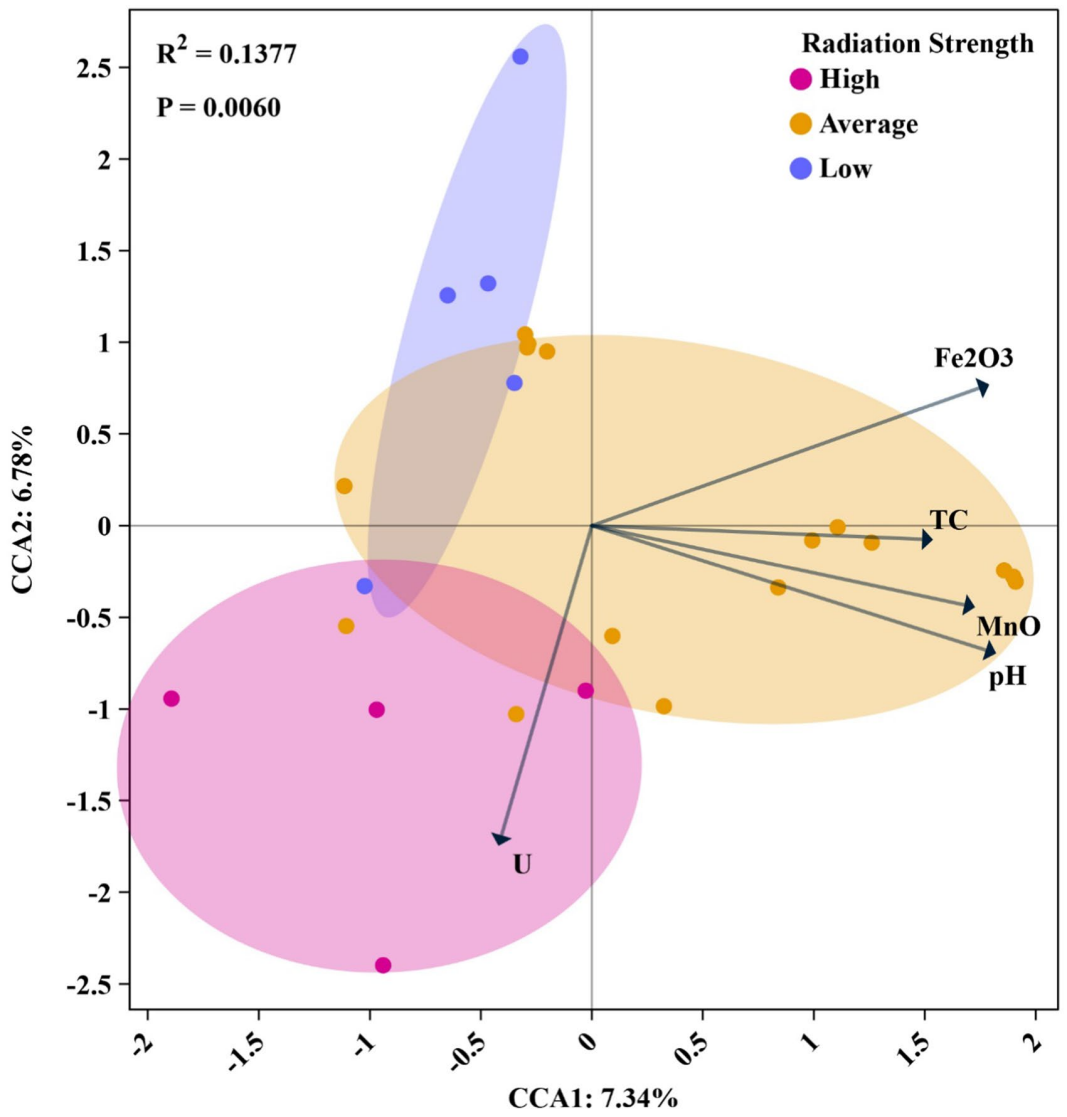
Canonical correspondence analysis (CCA) of microbial community composition constrained by environmental factors under different uranium contamination levels. The samples are grouped based on uranium contamination strength: high (10,000–18,000 mg/kg, pink), average (2,201–9,000 mg/kg, orange), and low (170–1,175 mg/kg, blue). Arrows indicated the direction and strength of environmental variables influencing microbial composition, including uranium (U), TC, MnO, pH, and Fe₂O₃. The analysis showed a significant correlation between microbial community structure and radiation strength (*R*^2^ = 0.1377, *p* = 0.0060), with the first and second CCA axes explaining 7.34 and 6.78% of the variance, respectively.

The results showed that uranium concentration strongly influenced microbial community composition, particularly in the high U group (Figure 4). In addition to U, other environmental factors, such as pH, Fe_2_O_3_, MnO and TC, appeared to significantly impact microbial community composition in the average U group, i.e., at the intermediate level of uranium contamination (Figure 4). Previous studies have shown that U levels greatly affected the assembly of microbial community. For example, Sutcliffe et al. (2017) reported that uranium influenced soil microbial community at concentrations from 0 to 4,000 mg/kg, and Mumtaz et al. (2018) found that uranium became a dominant factor in shaping microbial communities at the concentrations greater than 900 mg/kg. In our study, we proposed that within these uranium levels, i.e., 3,950 to 5,288 mg/kg in average-U, uranium might not be the sole factor determining community composition. Instead, other factors (pH, Fe_2_O_3_, TC, MnO), played a more significant role. However, in samples with concentrations exceeding 10,000 mg/kg, uranium became the primary factor shaping the microbial communities.

**Figure 5:**
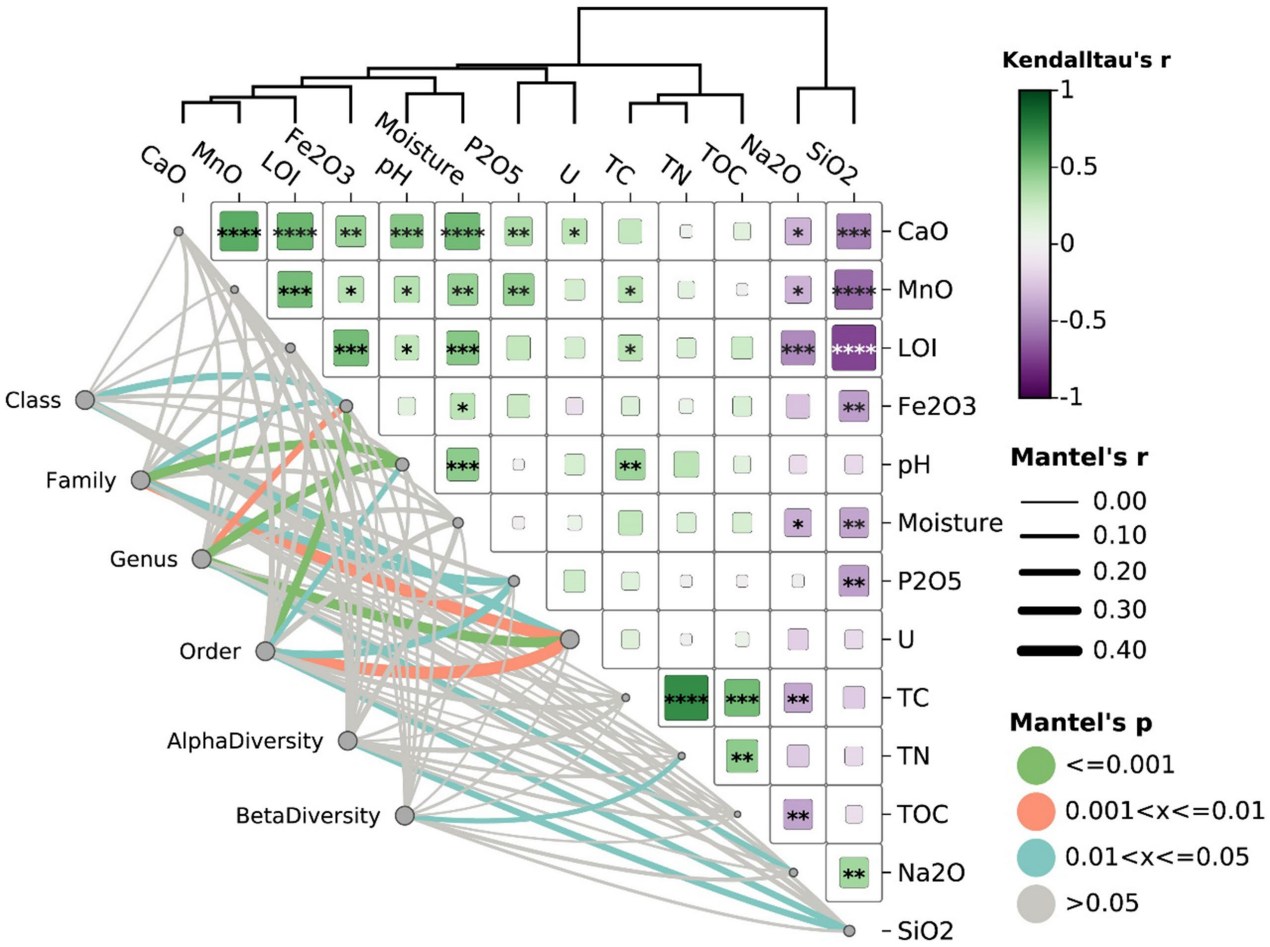
Correlation and Mantel test analysis of microbial diversity with physicochemical parameters (Euclidean distance). The heatmap on the right represents Kendall’s tau correlation coefficients (*r*) between environmental variables, with color intensity indicating the strength and direction of the correlation (green for positive, purple for negative). The significance of correlations was indicated by asterisks (*p ≤ 0.05, **p ≤ 0.01, ***p ≤ 0.001). The network on the left visualized Mantel’s test results, showing associations between microbial diversity metrics and physicochemical parameters. Line thickness reflects Mantel’s r, representing correlation strength, while line color represents significance levels (green for *p* ≤ 0.001, orange for 0.001 < *p* ≤ 0.01, blue for 0.01 < *p* ≤ 0.05, and gray for *p* > 0.05).

Furthermore, Mantel results showed that alpha diversity in all samples was positively correlated with SiO_2_ content (Figure 5, Mantel’s *r*-value = 0.208, *p*-value <0.05). As previously noted, SiO₂ serves as a key indicator of granite or rhyolite weathering, with higher SiO₂ concentrations corresponding to early-stage weathering. As weathering progresses and intensifies, it enhances the availability of trace elements within minerals or rocks, thereby facilitating microbial access and utilization (Fang et al., 2023; Zaharescu et al., 2019). Beta-diversity (microbial composition) was significantly associated with TN (Mantel’s *p*-value ≤0.05), suggesting the potential impact of nutrient availability on microbial communities. A total of 10 classes, 24 orders, 27 families, and 36 genera were found to be significantly associated with uranium contamination (details provided in Supplementary Table S4). For example, *Bauldia*, *Ellin6067* (a genus within the family Nitrosomonadaceae), Acidobacteriaceae, Gaiellales, Elsterales, Chroococcidiopsaceae, Roseiflexaceae, Chlamydiae, and Anaerolineae exhibited significant positive correlations with uranium concentrations (Supplementary Table S4, Mantel’s *r*-value >0, *p*-value <0.05). This suggested that these taxa may possess traits enabling them to resist radiation or thrive under elevated radiation levels. Conversely, taxa such as Planctomycetales, Methylopilaceae, *Modestobacter* and *Polaromonas* showed significant negative correlations with uranium concentrations (Supplementary Table S4, Mantel’s *r*-value <0, *p*-value <0.05), accounting for 15.5% of all identified taxa. This negative association suggested a potential vulnerability to uranium contamination or radiation stress. Although these U-related microbial communities showed significant associations with pH, Fe, and/or P_2_O_5_, the *r* values for these correlations were generally lower compared to those with uranium concentration, suggesting that uranium concentration levels significantly contributed to shaping certain microbial community composition.

### 3.4 Co-occurrence network of microbial communities

**Figure 6:**
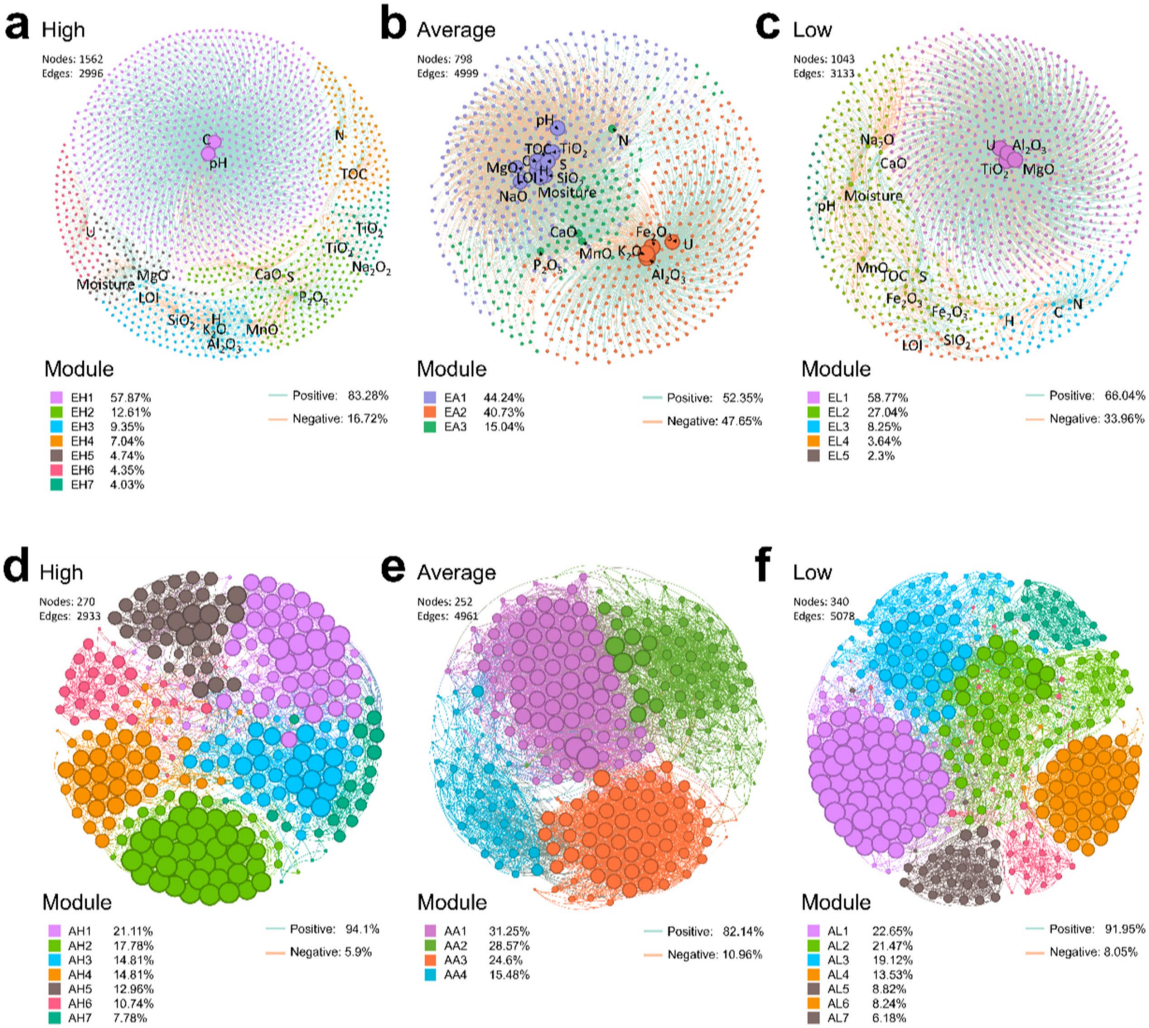
Correlation network between physicochemical factors and ASVs **(A–C)** and co-occurrence network between ASVs **(D–F)**. In panels **(D–F)**, the ASVs selected had a total relative abundance greater than 0.5%. All correlations were calculated using the Spearman method, with a threshold filter of *p* < 0.05 and r > 0.5. Green lines represent positive relationships, while red lines indicate negative relationships.

Correlation networks were constructed to assess the relative influence of physicochemical factors on microbial communities at ASV levels using the Spearman method (Figures 6A–C). The results revealed that positive correlations were more prevalent than negative ones across all groups, with values of 83.28, 52.35, and 66.04% in the high, average, and low uranium concentration groups, respectively (Figures 6A–C). Compared to the intermediate and low U groups, high-U conditions yield a large number of nodes (1,562) but relatively fewer edges (2,996). This configuration indicated a broad presence of taxa but weaker interconnections, with U-centered relationships confined to a single, relatively limited module (red color in Figure 6A), implying the presence of a cluster of taxa with metabolic activities specifically related to uranium (Figure 6A), such as *Scytonema* PCC-7110, unclassified Cyanobacteriales, Xanthobacteraceae, Kallotenuales, *Rubrobacter* and *Psychroglaciecola*. At the intermediate and low U levels, the correlation network between physicochemical parameters and ASVs was much denser and more integrative, with U no longer isolated from other parameters. Instead, it interacted with multiple modules and physicochemical parameters, suggesting that intermediate and relatively low U concentrations fostered a complex interplay among various physicochemical factors (Figure 6B), consistent with CCA results.

Similarly, co-occurrence networks of microbial communities were constructed to explore the interconnections between ASVs. The results showed a higher proportion of positive correlations across all groups with 94.1, 82.14, and 91.95%, correlations in the high, average, and low uranium concentration groups, respectively, suggesting that taxa favored cooperation over competition (Figures 6D–F). Interestingly, the edges were fewest in the high-U group (Figures 6A,D). Furthermore, the average degree (avgK), an indicator of network complexity, was lower in the high-U group compared to the other two groups (Table 1). In contrast, the average path distance (GD), representing the mean shortest path length between all node pairs in the network, was higher in the high-U group than in the other two groups. This indicated a simpler, more unstable network with limited microbial interactions, likely resulting from environmental constraints that restricted microbial co-occurrence (e.g., extreme conditions or resource scarcity). The reduced microbial interactions in the high-U group also suggested that those taxa developed niche-specific survival mechanisms. The less densely connected network is likely a result of more stochastic assembly processes (Sheng et al., 2021b).

**Table 1:** Topological properties of the correlation networks.

Networks^a^	U level	Node	Total links^b^	avgK^c^	GD^d^	ND^e^	Modularity
Correlation network	High	1,562	2,996	3.836	3.877	8	0.511
	Average	798	4,999	12.529	2.875	6	0.349
	Low	1,043	3,133	6.008	3.062	7	0.347
Co-occurrence network	High	270	2,933	21.726	3.012	6	0.688
	Average	252	4,961	39.373	2.406	6	0.53
	Low	340	5,078	29.871	2.949	7	0.667

a Correlation network: physicochemical factors and ASVs; Co-occurrence network: ASVs.

b Total links in the ecological network including both positive and negative links.

c Average degree, the average number of connections per node in the network.

d Average path distance, the average of the shortest path lengths between all pairs of nodes in the network.

e Network diameter, the maximum of the shortest path lengths between all pairs of nodes in the network.

### 3.5 Deterministic vs. stochastic assembly processes of microbial communities

**Figure 7:**
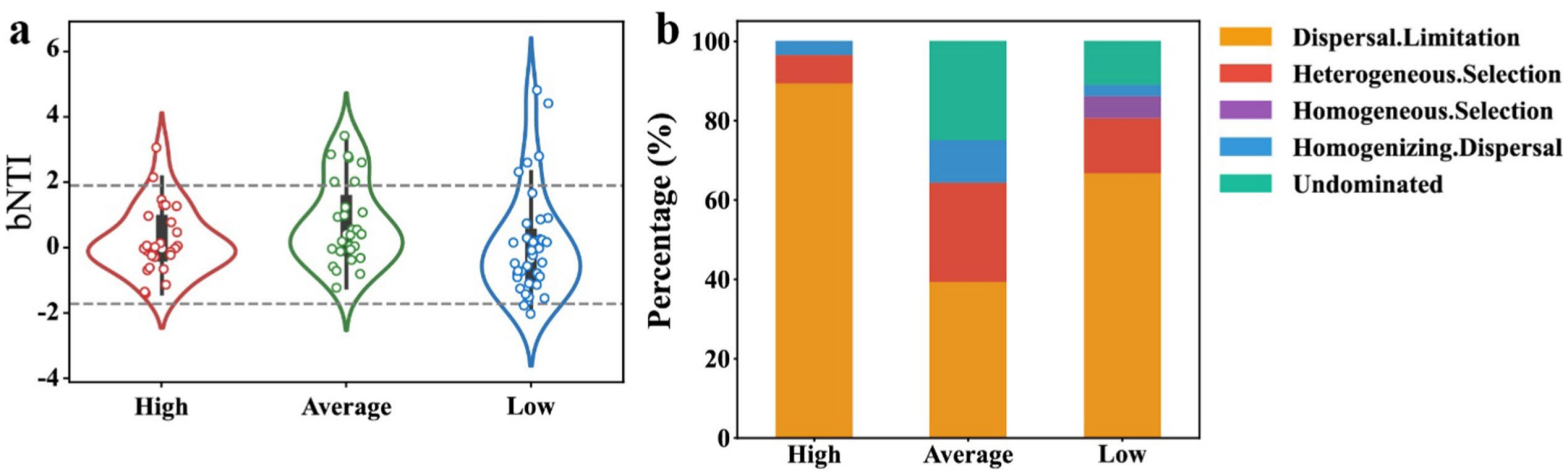
Analysis of stochastic and deterministic processes in microbial community assembly under different radiation levels. **(A)** *β*-nearest taxon index (βNTI) values for high, average, and low radiation strengths, with values outside the range of −2 to +2 indicating deterministic processes. **(B)** The relative contributions of different ecological processes, including dispersal limitation, heterogeneous selection, homogeneous selection, homogenizing dispersal, and undominated processes, across the uranium contamination strength categories.

Understanding the interplay between deterministic and stochastic processes in microbial community assembly is a key question, but poorly understood, in microbial ecology. One may anticipate intensive uranium contamination would lead to more deterministic assembly of microbial communities (Zhou and Ning, 2017). Interestingly, in the high-U group, community assembly was predominantly driven by dispersal limitation (90%), indicative of a stochastic process (Figure 7). In the average uranium concentration group, the proportion of stochastic processes reduced to ~55%, with heterogeneous selection, a deterministic process, accounting for ~20% and approximately ~25% attributed to undominated process, suggesting that selective pressures of environmental variations partially shaped the community. In the low uranium concentration group, in addition to dispersal limitation, heterogeneous selection accounted for ~10% and homogeneous selection accounted for 4%, with the remaining processes categorized as undominated. The increased homogeneous selection suggested a convergence towards a common adaptive strategy, driven by reduced selection pressures and more consistent environmental conditions. These results highlighted that as uranium contamination increased up to 10,000 mg/kg, community assembly became more driven by dispersal limitation, implying that the communities were more isolated, potentially reflecting a less stable ecosystem (Sheng et al., 2021b,c). This finding aligned with the co-occurrence network results, which showed the least interconnected microbial associations in the high-U group (Figure 6).

At some heavy metal-contaminated sites, such as those with chromium (Cr) in soils, the assembly processes of bacterial communities shifted from stochastic to deterministic processes with elevated contamination (Liu et al., 2023). Similarly, in uranium-contaminated underground water with relatively low concentrations (~20 mg/L), deterministic processes dominated due to the selective pressures on microbial communities (Michael et al., 2024; Ning et al., 2024). However, Yu et al. (2024) found that in heavy metal (especially cadmium Cd) contaminated marine sediments, the archaeal community was shaped by stochastic processes. Moreover, in deep soil layers where nutrients were largely limited or severely contaminated by petroleum hydrocarbons, the microbial communities became more stochastically assembly (primarily dispersal limitation) (Sheng et al., 2021c). Our results consistently indicated that uranium concentration differentially influenced microbial community assembly, with dispersal limitation dominating at extremely high uranium concentrations, while a transition to deterministic processes occurred at moderate to low concentrations. This study highlights the complexity of microbial communities in uranium-contaminated environments, where diverse survival strategies may be employed to cope with extreme conditions.

## 4 Conclusion

This study investigates the influence of uranium contamination on microbial community composition in mine tailing environments. Mining operations and remediation efforts resulted in geochemical heterogeneity across different sites, such as uranium concentration, pH, Fe₂O₃, and P₂O₅. Despite these varied conditions, microbial communities remained adaptable, with Proteobacteria and Cyanobacteria dominating uranium-enriched environments. Multivariate analyses identified uranium concentration as the primary driver of microbial community structure, followed by other factors like Fe₂O₃ and pH. Network analyses revealed simpler, less interconnected microbial networks under high uranium conditions (>10,000 mg/kg), while intermediate and relatively low uranium levels supported more complex assemblages. The study showed that in high-uranium environments, stochastic processes such as dispersal limitation dominated community assembly, whereas deterministic processes, including both heterogeneous and homogeneous selection, were more significant in those environments with moderate to low uranium concentrations. There might be a threshold that determines how microbial communities respond to the high uranium concentrations. At lower concentrations, cooperation may prevail, while at higher concentrations, microbes may shift to a competitive “fight for their own” strategy. These findings highlight the complex interplay between uranium contamination and microbial ecology, offering insights into potential bioremediation strategies and the resilience of microbial communities under heavy metal and radiation stress.

## Data Availability

The data presented in the study were deposited in the NCBI repository, accession number PRJNA1200928.
