# Supplementary material for: Uranium contamination mediating soil and ore microbial community assembly at four mining sites, South China
Source: Front Microbiol. 2025 Feb 19;16:1553072. doi: 10.3389/fmicb.2025.1553072 (PMC11879985; doi:10.3389/fmicb.2025.1553072)
Supplement: Supplementary file 1 [file Supplementary_file_1.docx]

**SUPPLEMENTAL INFORMATION**

Uranium contamination mediating soil and ore microbial community assembly at four mining sites, South China

Hongyu Chen^1^, Yizhi Sheng^1,2*^, Shuaidi Wang^1,2^, Yu Chen^3^, Zhiyuan Qiao^4^, Huaming Guo^2,4^, Hailiang Dong^1,2*^

^1^Center for Geomicrobiology and Biogeochemistry Research, State Key Laboratory of Biogeology and Environmental Geology, China University of Geosciences, Beijing 100083, China

^2^Frontiers Science Center for Deep-time Digital Earth, China University of Geosciences, Beijing 100083, China

^3^School of Environment, Tsinghua University, Beijing 100084, China

^4^MOE Key Laboratory of Groundwater Circulation and Evolution & School of Water Resources and Environment, China University of Geosciences, Beijing 100083,

China

*** Correspondence:**Yizhi Sheng, Hailiang Dong
shengyz@cugb.edu.cn & [dongh@cugb.edu.cn](mailto:dongh@cugb.edu.cn)

| Sample | U (mg/kg) | SiO_2_ (wt%) | TiO_2_ (wt%) | Al_2_O_3_ (wt%) | Fe_2_O_3_ (wt%) | MnO (wt%) | MgO (wt%) | CaO (wt%) | Na_2_O (wt%) | K_2_O (wt%) | P_2_O_5_ (wt%) |
| --- | --- | --- | --- | --- | --- | --- | --- | --- | --- | --- | --- |
| XC-015 | 3399 | 69.43 | 0.93 | 12.41 | 5.03 | 0.06 | 1.16 | 2.22 | 0.97 | 3.00 | 0.16 |
| XC-030 | 3643 | 64.29 | 1.02 | 12.68 | 6.64 | 0.09 | 1.51 | 3.17 | 0.90 | 3.21 | 0.23 |
| XC-050 | 3950 | 63.59 | 0.89 | 12.56 | 6.26 | 0.09 | 1.27 | 4.28 | 1.47 | 3.33 | 0.17 |
| XC-070 | 4126 | 63.42 | 0.91 | 13.35 | 6.47 | 0.08 | 1.50 | 3.61 | 1.64 | 3.36 | 0.21 |
| XC-090 | 4204 | 42.80 | 0.49 | 12.36 | 8.43 | 0.80 | 0.00 | 21.90 | 0.50 | 2.29 | 0.36 |
| XC-110 | 4041 | 2.66 | 0.04 | 9.56 | 10.23 | 1.56 | 0.00 | 61.71 | 0.00 | 0.09 | 0.41 |
| XC-130 | 4113 | 1.37 | 0.02 | 8.59 | 9.94 | 1.64 | 0.00 | 61.93 | 0.54 | 0.03 | 0.37 |
| XC-150 | 4454 | 1.14 | 0.01 | 8.53 | 9.49 | 1.81 | 0.00 | 63.56 | 0.00 | 0.01 | 0.38 |
| XC-170 | 5099 | 54.24 | 0.41 | 16.28 | 6.26 | 0.14 | 0.56 | 8.84 | 0.58 | 3.79 | 0.28 |
| XC-190 | 5288 | 61.44 | 0.32 | 19.39 | 3.24 | 0.07 | 0.54 | 3.13 | 0.54 | 4.33 | 0.12 |
| XC-210 | 5510 | 52.92 | 0.53 | 16.73 | 6.74 | 0.11 | 0.74 | 7.82 | 0.45 | 3.51 | 0.28 |
| ZSX-1-1 | 6785 | 65.85 | 0.14 | 20.74 | 1.35 | 0.07 | 0.68 | 0.79 | 0.54 | 4.35 | 0.03 |
| ZSX-1-2 | 170 | 66.67 | 0.11 | 20.69 | 1.06 | 0.07 | 0.61 | 0.17 | 0.65 | 4.72 | 0.02 |
| GX2 | 900 | 66.80 | 0.58 | 15.75 | 5.22 | 0.04 | 0.90 | 0.41 | 1.95 | 3.76 | 0.22 |
| GX3 | 1100 | 64.03 | 0.45 | 16.02 | 5.84 | 0.05 | 0.60 | 1.20 | 2.30 | 3.98 | 0.39 |
| GX4 | 16000 | 66.15 | 0.46 | 12.49 | 3.25 | 0.07 | 0.94 | 4.76 | 0.73 | 2.87 | 0.13 |
| GX5 | 6800 | 71.36 | 0.63 | 13.60 | 3.12 | 0.08 | 0.72 | 1.96 | 0.94 | 2.43 | 0.14 |
| GX7 | 1175 | 74.29 | 0.48 | 13.37 | 2.25 | 0.04 | 0.55 | 0.57 | 1.58 | 3.37 | 0.14 |
| XS2 | 2700 | 62.32 | 0.45 | 13.35 | 4.25 | 0.86 | 0.64 | 7.78 | 1.35 | 3.16 | 0.35 |
| XS3 | 700 | 62.81 | 0.90 | 19.34 | 7.04 | 0.07 | 0.34 | 0.16 | 0.28 | 2.15 | 0.14 |
| XS4 | 18000 | 44.47 | 0.54 | 19.39 | 3.40 | 0.14 | 0.52 | 17.27 | 0.49 | 4.81 | 0.95 |
| XS5 | 9000 | 51.63 | 0.53 | 24.91 | 3.05 | 0.16 | 0.59 | 3.60 | 1.76 | 5.55 | 1.46 |
| XS6 | 10000 | 76.25 | 0.21 | 12.07 | 1.77 | 0.07 | 0.17 | 1.51 | 1.58 | 2.03 | 0.68 |
| XS7 | 2201 | 52.70 | 0.59 | 28.19 | 3.93 | 0.08 | 0.75 | 0.59 | 1.64 | 5.96 | 0.28 |
| XS8 | 11750 | 49.12 | 0.50 | 28.77 | 3.83 | 0.14 | 0.56 | 1.38 | 0.25 | 6.70 | 0.49 |

**Table S1** Geochemical parameters of samples in this work

**Table S1. continued** Geochemical parameters of samples in this work

| Sample | LOI | TOC (ppm) | TN (%) | TC (%) | H (%) | S (%) | Moisture (%) | pH |
| --- | --- | --- | --- | --- | --- | --- | --- | --- |
| XC-015 | 4.09 | 1674.15 | 0.02 | 0.13 | 0.499 | 1.059 | 9.49 | 5.43 |
| XC-030 | 5.55 | 33.37 | 0.01 | 0.07 | 0.313 | 0.263 | 8.88 | 4.98 |
| XC-050 | 5.20 | 98.62 | 0.01 | 0.04 | 0.25 | 0.377 | 9.25 | 4.66 |
| XC-070 | 5.26 | 188.27 | 0.01 | 0.03 | 0.249 | 0.332 | 9.42 | 5.19 |
| XC-090 | 9.36 | 1016.83 | 0.01 | 0.11 | 0.671 | 2.432 | 21.64 | 6.3 |
| XC-110 | 14.21 | 12535.18 | 0.03 | 2.13 | 1.928 | 6.131 | 30.62 | 7.29 |
| XC-130 | 15.83 | 27928.38 | 0.03 | 2.03 | 2.229 | 5.285 | 44.03 | 7.56 |
| XC-150 | 15.35 | 22475.84 | 0.03 | 2.74 | 2.095 | 4.897 | 54.14 | 7.61 |
| XC-170 | 8.10 | 825.43 | 0.01 | 0.07 | 0.355 | 1.213 | 12.03 | 7.6 |
| XC-190 | 6.32 | 1148.58 | 0.02 | 0.16 | 0.364 | 0.872 | 11.05 | 7.53 |
| XC-210 | 9.37 | 188.40 | 0.01 | 0.07 | 0.332 | 0.615 | 12.94 | 7.34 |
| ZSX-1-1 | 4.90 | 1007.41 | 0.01 | 0.06 | 0.248 | 0.029 | 10.25 | 3.98 |
| ZSX-1-2 | 4.74 | 175.90 | 0.01 | 0.03 | 0.097 | 0.074 | 11.32 | 4.63 |
| GX2 | 4.72 | 262.41 | 0.01 | 0.05 | 0.173 | 0.134 | 3.00 | 3.93 |
| GX3 | 5.05 | 381.36 | 0.01 | 0.05 | 0.139 | 0.146 | 5.90 | 4.49 |
| GX4 | 7.59 | 2095.86 | 0.04 | 0.89 | 0.225 | 0.064 | 11.85 | 7.67 |
| GX5 | 4.27 | 361.73 | 0.02 | 0.41 | 0.126 | 0.041 | 8.94 | 7.91 |
| GX7 | 3.44 | 1354.38 | 0.03 | 0.31 | 0.148 | 0.044 | 6.53 | 6.86 |
| XS2 | 4.93 | 86.60 | 0.03 | 0.29 | 0.236 | 0.697 | 11.83 | 8.26 |
| XS3 | 7.19 | 12801.08 | 0.06 | 1.1 | 0.7 | 0.227 | 7.14 | 4.25 |
| XS4 | 7.22 | 206.40 | 0.01 | 0.07 | 0.493 | 0.732 | 1.04 | 4.99 |
| XS5 | 6.45 | 77.18 | 0.02 | 0.22 | 0.673 | 0.413 | 11.13 | 5.57 |
| XS6 | 2.67 | 613.30 | 0.01 | 0.07 | 0.198 | 0.126 | 2.32 | 4.33 |
| XS7 | 4.91 | 104.90 | 0.02 | 0.06 | 0.54 | 0.16 | 8.04 | 3.55 |
| XS8 | 7.34 | 1975.47 | 0.03 | 0.26 | 0.721 | 0.227 | 5.45 | 4.08 |

**Table S2.** Read counts at each step of 16S QIIME2 quality control

| **Sample** | Input | Filtered | Percentage of input passing filter | Denoised | Merged | Percentage of input merged | Non-chimeric | Percentage of input non-chimeric |
| --- | --- | --- | --- | --- | --- | --- | --- | --- |
| XC-015 | 122303 | 101567 | 83.05 | 96918 | 90919 | 74.34 | 89257 | 72.98 |
| XC-030 | 139194 | 115847 | 83.23 | 111981 | 106768 | 76.7 | 105199 | 75.58 |
| XC-050 | 78528 | 65352 | 83.22 | 62844 | 60505 | 77.05 | 57285 | 72.95 |
| XC-070 | 94556 | 77924 | 82.41 | 76071 | 73414 | 77.64 | 72937 | 77.14 |
| XC-090 | 73870 | 61030 | 82.62 | 60214 | 59136 | 80.05 | 58709 | 79.48 |
| XC-110 | 97420 | 79984 | 82.1 | 79104 | 78319 | 80.39 | 78171 | 80.24 |
| XC-130 | 78008 | 64000 | 82.04 | 63282 | 62534 | 80.16 | 62510 | 80.13 |
| XC-150 | 86606 | 71435 | 82.48 | 70496 | 69674 | 80.45 | 69665 | 80.44 |
| XC-170 | 125643 | 102003 | 81.18 | 99766 | 96961 | 77.17 | 96131 | 76.51 |
| XC-190 | 151954 | 121521 | 79.97 | 116586 | 110936 | 73.01 | 108089 | 71.13 |
| XC-210 | 110367 | 90136 | 81.67 | 86366 | 82015 | 74.31 | 81657 | 73.99 |
| ZSX-1-1 | 157752 | 130834 | 82.94 | 124269 | 113084 | 71.68 | 109209 | 69.23 |
| ZSX-1-2 | 163984 | 136736 | 83.38 | 128608 | 114314 | 69.71 | 110763 | 67.55 |
| GX2 | 131855 | 108000 | 81.91 | 103893 | 97623 | 74.04 | 95119 | 72.14 |
| GX3 | 137176 | 113440 | 82.7 | 110692 | 107742 | 78.54 | 104766 | 76.37 |
| GX4 | 189302 | 157832 | 83.38 | 145506 | 125631 | 66.37 | 123929 | 65.47 |
| GX5 | 151122 | 125305 | 82.92 | 113961 | 96930 | 64.14 | 95292 | 63.06 |
| GX7 | 100250 | 83563 | 83.35 | 80690 | 77542 | 77.35 | 75279 | 75.09 |
| XS2 | 145543 | 121143 | 83.24 | 116975 | 111401 | 76.54 | 108016 | 74.22 |
| XS3 | 163970 | 136476 | 83.23 | 131111 | 123669 | 75.42 | 119233 | 72.72 |
| XS4 | 130635 | 108529 | 83.08 | 104906 | 100696 | 77.08 | 97658 | 74.76 |
| XS5 | 156872 | 130825 | 83.4 | 126191 | 119486 | 76.17 | 118117 | 75.3 |
| XS6 | 153422 | 125467 | 81.78 | 122378 | 117241 | 76.42 | 111569 | 72.72 |
| XS7 | 139247 | 110048 | 79.03 | 108343 | 101115 | 72.62 | 99107 | 71.17 |
| XS8 | 170192 | 140780 | 82.72 | 134979 | 125080 | 73.49 | 119615 | 70.28 |

**Table S3.** Alpha diversity of samples

| Sample | Observed | Chao1 | se.chao1* | ACE | se.ACE* | Shannon | Simpson | InvSimpson | Fisher |
| --- | --- | --- | --- | --- | --- | --- | --- | --- | --- |
| XC-015 | 1299 | 1419.41 | 21.79 | 1415.86 | 17.16 | 5.66 | 0.98 | 62.82 | 310.78 |
| XC-030 | 1266 | 1420.07 | 25.79 | 1420.07 | 17.42 | 5.52 | 0.98 | 60.48 | 300.50 |
| XC-050 | 675 | 734.90 | 15.59 | 730.87 | 12.69 | 4.13 | 0.91 | 10.60 | 134.83 |
| XC-070 | 925 | 992.50 | 15.40 | 994.66 | 14.37 | 4.69 | 0.94 | 16.96 | 200.55 |
| XC-090 | 664 | 690.56 | 9.87 | 681.25 | 12.59 | 5.12 | 0.98 | 65.71 | 132.10 |
| XC-110 | 428 | 475.12 | 17.62 | 454.73 | 10.51 | 3.75 | 0.89 | 9.40 | 76.91 |
| XC-130 | 552 | 566.17 | 6.76 | 561.15 | 11.37 | 4.27 | 0.93 | 15.00 | 105.06 |
| XC-150 | 445 | 462.45 | 7.36 | 462.28 | 10.34 | 3.89 | 0.93 | 13.63 | 80.65 |
| XC-170 | 984 | 1050.02 | 15.09 | 1055.68 | 15.40 | 5.46 | 0.99 | 72.72 | 217.01 |
| XC-190 | 978 | 1116.62 | 25.58 | 1121.91 | 16.05 | 5.04 | 0.97 | 33.06 | 215.32 |
| XC-210 | 1215 | 1306.02 | 17.96 | 1317.67 | 17.28 | 5.92 | 0.99 | 124.26 | 284.83 |
| ZSX-1-1 | 1279 | 1432.14 | 25.73 | 1429.48 | 17.33 | 5.30 | 0.97 | 32.65 | 304.54 |
| ZSX-1-2 | 1511 | 1731.43 | 32.18 | 1717.07 | 18.72 | 5.56 | 0.98 | 48.71 | 379.26 |
| GX2 | 685 | 729.51 | 12.58 | 731.61 | 13.06 | 5.12 | 0.99 | 69.37 | 137.33 |
| GX3 | 500 | 552.92 | 14.75 | 564.08 | 11.66 | 4.38 | 0.96 | 27.28 | 93.02 |
| GX4 | 3053 | 3449.47 | 41.67 | 3420.84 | 24.97 | 7.40 | 1.00 | 789.39 | 1004.06 |
| GX5 | 2685 | 2936.91 | 31.07 | 2928.44 | 22.96 | 7.19 | 1.00 | 412.81 | 834.49 |
| GX7 | 550 | 578.11 | 9.38 | 586.91 | 11.90 | 4.44 | 0.94 | 16.23 | 104.59 |
| XS2 | 902 | 1021.35 | 25.11 | 997.97 | 15.19 | 5.26 | 0.97 | 37.79 | 194.22 |
| XS3 | 1086 | 1200.61 | 22.44 | 1191.24 | 16.33 | 5.60 | 0.99 | 95.45 | 246.31 |
| XS4 | 991 | 1091.56 | 19.68 | 1101.95 | 15.17 | 4.70 | 0.95 | 20.85 | 218.99 |
| XS5 | 1560 | 1755.00 | 28.83 | 1759.69 | 19.04 | 5.81 | 0.98 | 55.45 | 395.69 |
| XS6 | 530 | 587.59 | 16.01 | 583.39 | 11.53 | 3.98 | 0.93 | 14.69 | 99.92 |
| XS7 | 314 | 314.22 | 0.52 | 315.78 | 8.83 | 3.83 | 0.94 | 16.76 | 52.88 |
| XS8 | 1009 | 1187.14 | 31.55 | 1154.27 | 15.66 | 5.07 | 0.97 | 39.65 | 224.09 |

*se: standard error

**Table S4.** Uranium sensitive taxon which had significant correlations with uranium concentrations

| **Taxon** | **p-value** | **r** | **z-score** |
| --- | --- | --- | --- |
| Bacteria;Verrucomicrobiota;Chlamydiae | 0.03 | 0.14 | 2.23 |
| Bacteria;Verrucomicrobiota;Verrucomicrobiae;Methylacidiphilales | 0.04 | 0.23 | 1.98 |
| Bacteria;Verrucomicrobiota;Verrucomicrobiae;UA11 | 0.01 | 0.23 | 2.21 |
| Bacteria;Verrucomicrobiota;Verrucomicrobiae;Opitutales;Puniceicoccaceae | 0.03 | 0.19 | 1.82 |
| Bacteria;Verrucomicrobiota;Verrucomicrobiae;Opitutales;Opitutaceae; | 0.03 | 0.21 | 1.91 |
| Bacteria;Verrucomicrobiota;Verrucomicrobiae;Opitutales;Puniceicoccaceae; | 0.01 | 0.20 | 2.03 |
| Bacteria;Bacteroidota; | 0.05 | -0.18 | -1.94 |
| Bacteria;Bacteroidota;Kryptonia | 0.00 | 0.35 | 3.16 |
| Bacteria;Bacteroidota;Bacteroidia;Cytophagales | 0.03 | 0.18 | 2.05 |
| Bacteria;Bacteroidota;Bacteroidia;Chitinophagales;Chitinophagaceae;Heliimonas | 0.04 | -0.21 | -2.03 |
| Bacteria;Bacteroidota;Bacteroidia;Chitinophagales;Chitinophagaceae;Lacibacter | 0.03 | 0.22 | 1.94 |
| Bacteria;Bacteroidota;Bacteroidia;Cytophagales;Cytophagaceae;Sporocytophaga | 0.02 | 0.20 | 1.93 |
| Bacteria;Bacteroidota;Bacteroidia;Cytophagales;Microscillaceae;OLB12 | 0.01 | 0.22 | 2.03 |
| Bacteria;Bacteroidota;Bacteroidia;Cytophagales;Spirosomaceae;Huanghella | 0.01 | 0.31 | 2.79 |
| Bacteria;Acidobacteriota;Acidobacteriae;Acidobacteriales | 0.02 | 0.28 | 2.32 |
| Bacteria;Acidobacteriota;Acidobacteriae;Subgroup 2 | 0.02 | 0.23 | 2.08 |
| Bacteria;Acidobacteriota;Blastocatellia;Pyrinomonadales | 0.04 | 0.26 | 2.06 |
| Bacteria;Acidobacteriota;Holophagae;Holophagales | 0.04 | 0.18 | 1.88 |
| Bacteria;Acidobacteriota;Acidobacteriae;Acidobacteriales;Acidobacteriaceae (Subgroup 1) | 0.04 | 0.22 | 1.93 |
| Bacteria;Acidobacteriota;Acidobacteriae;Acidobacteriales;Acidobacteriaceae (Subgroup 1);Edaphobacter | 0.01 | 0.24 | 2.21 |
| Bacteria;Acidobacteriota;Acidobacteriae;Acidobacteriales;Acidobacteriaceae (Subgroup 1);Occallatibacter | 0.01 | 0.25 | 2.36 |
| Bacteria;Acidobacteriota;Acidobacteriae;Acidobacteriales;Acidobacteriaceae (Subgroup 1);Terracidiphilus | 0.04 | 0.23 | 2.04 |
| Bacteria;Planctomycetota;Planctomycetes;Planctomycetales | 0.01 | 0.38 | 2.80 |
| Bacteria;Planctomycetota;Planctomycetes;Planctomycetales; | 0.01 | -0.24 | -2.28 |
| Bacteria;Planctomycetota;Planctomycetes;Planctomycetales;uncultured | 0.03 | 0.32 | 2.34 |
| Bacteria;Cyanobacteria;Cyanobacteriia | 0.00 | 0.35 | 4.31 |
| Bacteria;Cyanobacteria;Cyanobacteriia;Cyanobacteriales | 0.00 | 0.35 | 3.37 |
| Bacteria;Cyanobacteria;Cyanobacteriia;Cyanobacteriales; | 0.02 | 0.28 | 2.27 |
| Bacteria;Cyanobacteria;Cyanobacteriia;Cyanobacteriales;Chamaesiphonaceae | 0.01 | 0.33 | 2.92 |
| Bacteria;Cyanobacteria;Cyanobacteriia;Cyanobacteriales;Chroococcidiopsaceae | 0.05 | 0.20 | 1.90 |
| Bacteria;Cyanobacteria;Cyanobacteriia;Cyanobacteriales;Coleofasciculaceae; | 0.01 | -0.23 | -2.34 |
| Bacteria;Chloroflexi;Anaerolineae | 0.03 | 0.18 | 2.15 |
| Bacteria;Chloroflexi;Chloroflexia | 0.00 | 0.35 | 3.57 |
| Bacteria;Chloroflexi;P2-11E | 0.00 | 0.52 | 4.39 |
| Bacteria;Chloroflexi;Chloroflexia;Chloroflexales | 0.00 | 0.46 | 4.23 |
| Bacteria;Chloroflexi;Chloroflexia;Kallotenuales | 0.03 | 0.26 | 2.12 |
| Bacteria;Chloroflexi;Dehalococcoidia; | 0.04 | -0.17 | -1.82 |
| Bacteria;Chloroflexi;Dehalococcoidia;SAR202 clade | 0.05 | 0.22 | 1.91 |
| Bacteria;Chloroflexi;Ktedonobacteria;B10-SB3A | 0.04 | 0.17 | 1.82 |
| Bacteria;Chloroflexi;Chloroflexia;Chloroflexales;Roseiflexaceae | 0.02 | 0.27 | 2.54 |
| Bacteria;Chloroflexi;Chloroflexia;Kallotenuales;AKIW781 | 0.03 | 0.27 | 2.20 |
| Bacteria;Chloroflexi;Chloroflexia;Chloroflexales;Roseiflexaceae;uncultured | 0.02 | 0.27 | 2.48 |
| Bacteria;Chloroflexi;Ktedonobacteria;Ktedonobacterales;Ktedonobacteraceae;FCPS473 | 0.01 | -0.22 | -2.38 |
| Bacteria;Actinobacteriota;Acidimicrobiia | 0.03 | 0.26 | 2.26 |
| Bacteria;Actinobacteriota;Coriobacteriia | 0.01 | 0.20 | 2.06 |
| Bacteria;Actinobacteriota;Thermoleophilia | 0.00 | 0.35 | 3.41 |
| Bacteria;Actinobacteriota;Acidimicrobiia;Acidimicrobiales | 0.03 | 0.20 | 1.94 |
| Bacteria;Actinobacteriota;Acidimicrobiia;IMCC26256 | 0.03 | 0.26 | 2.12 |
| Bacteria;Actinobacteriota;Actinobacteria;Catenulisporales | 0.01 | 0.21 | 2.26 |
| Bacteria;Actinobacteriota;Coriobacteriia;CG2-30-50-142 | 0.01 | 0.20 | 1.97 |
| Bacteria;Actinobacteriota;Thermoleophilia;Gaiellales | 0.02 | 0.26 | 2.43 |
| Bacteria;Actinobacteriota;Acidimicrobiia;Actinomarinales; | 0.05 | 0.18 | 1.66 |
| Bacteria;Actinobacteriota;Actinobacteria;Catenulisporales;Actinospicaceae | 0.00 | 0.24 | 2.43 |
| Bacteria;Actinobacteriota;Actinobacteria;Corynebacteriales; | 0.03 | 0.22 | 1.92 |
| Bacteria;Actinobacteriota;Actinobacteria;Corynebacteriales;Corynebacteriaceae | 0.03 | 0.15 | 1.94 |
| Bacteria;Actinobacteriota;Actinobacteria;Corynebacteriales;Nocardiaceae | 0.01 | 0.31 | 2.66 |
| Bacteria;Actinobacteriota;Actinobacteria;Frankiales;Acidothermaceae | 0.04 | 0.21 | 1.88 |
| Bacteria;Actinobacteriota;Actinobacteria;Frankiales;Geodermatophilaceae | 0.01 | -0.24 | -2.37 |
| Bacteria;Actinobacteriota;Actinobacteria;Micrococcales;Intrasporangiaceae | 0.04 | -0.22 | -2.04 |
| Bacteria;Actinobacteriota;Thermoleophilia;Gaiellales;uncultured | 0.02 | 0.26 | 2.58 |
| Bacteria;Actinobacteriota;Actinobacteria;Corynebacteriales;Nocardiaceae;Nocardia | 0.01 | 0.29 | 2.76 |
| Bacteria;Actinobacteriota;Actinobacteria;Frankiales;Geodermatophilaceae;Modestobacter | 0.03 | -0.22 | -2.12 |
| Bacteria;Actinobacteriota;Actinobacteria;Micrococcales;Intrasporangiaceae; | 0.04 | -0.21 | -2.10 |
| Bacteria;Actinobacteriota;Actinobacteria;Micrococcales;Microbacteriaceae;Microbacterium | 0.01 | 0.31 | 2.78 |
| Bacteria;Actinobacteriota;Actinobacteria;Micrococcales;Microbacteriaceae;Parafrigoribacterium | 0.03 | 0.22 | 1.91 |
| Bacteria;Proteobacteria;Alphaproteobacteria;Elsterales | 0.01 | 0.30 | 2.89 |
| Bacteria;Proteobacteria;Gammaproteobacteria;Beggiatoales | 0.03 | 0.23 | 2.06 |
| Bacteria;Proteobacteria;Gammaproteobacteria;Coxiellales | 0.04 | 0.22 | 2.06 |
| Bacteria;Proteobacteria;Gammaproteobacteria;Diplorickettsiales | 0.02 | 0.15 | 2.37 |
| Bacteria;Proteobacteria;Gammaproteobacteria;Gammaproteobacteria Incertae Sedis | 0.04 | 0.18 | 2.12 |
| Bacteria;Proteobacteria;Alphaproteobacteria;Defluviicoccales;uncultured | 0.02 | 0.22 | 2.05 |
| Bacteria;Proteobacteria;Alphaproteobacteria;Elsterales;uncultured | 0.01 | 0.30 | 2.91 |
| Bacteria;Proteobacteria;Alphaproteobacteria;Rhizobiales;Methylopilaceae | 0.01 | -0.22 | -2.16 |
| Bacteria;Proteobacteria;Alphaproteobacteria;Rhizobiales;Rhizobiales Incertae Sedis | 0.03 | 0.24 | 2.16 |
| Bacteria;Proteobacteria;Gammaproteobacteria;Burkholderiales;Nitrosomonadaceae | 0.01 | 0.32 | 2.96 |
| Bacteria;Proteobacteria;Gammaproteobacteria;Burkholderiales;Rhodocyclaceae | 0.03 | 0.18 | 1.77 |
| Bacteria;Proteobacteria;Gammaproteobacteria;Burkholderiales;Sulfuricellaceae | 0.02 | -0.18 | -2.21 |
| Bacteria;Proteobacteria;Gammaproteobacteria;Pseudomonadales;Moraxellaceae | 0.02 | 0.24 | 2.16 |
| Bacteria;Proteobacteria;Gammaproteobacteria;Steroidobacterales;Woeseiaceae | 0.00 | 0.26 | 2.35 |
| Bacteria;Proteobacteria;Alphaproteobacteria;Acetobacterales;Acetobacteraceae;Acidicaldus | 0.05 | 0.22 | 1.94 |
| Bacteria;Proteobacteria;Alphaproteobacteria;Acetobacterales;Acetobacteraceae;Craurococcus-Caldovatus | 0.04 | 0.18 | 1.83 |
| Bacteria;Proteobacteria;Alphaproteobacteria;Acetobacterales;Acetobacteraceae;Rhodovastum | 0.04 | 0.19 | 1.71 |
| Bacteria;Proteobacteria;Alphaproteobacteria;Azospirillales;Azospirillaceae; | 0.01 | 0.21 | 2.12 |
| Bacteria;Proteobacteria;Alphaproteobacteria;Rhizobiales;Beijerinckiaceae;Methylocella | 0.02 | 0.19 | 1.90 |
| Bacteria;Proteobacteria;Alphaproteobacteria;Rhizobiales;Devosiaceae;Arsenicitalea | 0.03 | 0.19 | 1.86 |
| Bacteria;Proteobacteria;Alphaproteobacteria;Rhizobiales;Methyloligellaceae;uncultured | 0.02 | 0.23 | 2.11 |
| Bacteria;Proteobacteria;Alphaproteobacteria;Rhizobiales;Rhizobiales Incertae Sedis;Bauldia | 0.04 | 0.22 | 2.07 |
| Bacteria;Proteobacteria;Alphaproteobacteria;Rhizobiales;Xanthobacteraceae;Nitrobacter | 0.04 | 0.18 | 1.66 |
| Bacteria;Proteobacteria;Gammaproteobacteria;Burkholderiales;Comamonadaceae;Polaromonas | 0.05 | -0.21 | -2.04 |
| Bacteria;Proteobacteria;Gammaproteobacteria;Burkholderiales;Hydrogenophilaceae;Hydrogenophilus | 0.00 | 0.25 | 2.52 |
| Bacteria;Proteobacteria;Gammaproteobacteria;Burkholderiales;Hydrogenophilaceae;uncultured | 0.03 | -0.21 | -2.17 |
| Bacteria;Proteobacteria;Gammaproteobacteria;Burkholderiales;Nitrosomonadaceae;Ellin6067 | 0.00 | 0.43 | 3.53 |
| Bacteria;Proteobacteria;Gammaproteobacteria;Burkholderiales;Rhodocyclaceae; | 0.02 | 0.20 | 1.87 |
| Bacteria;Proteobacteria;Gammaproteobacteria;Burkholderiales;Sulfuricellaceae;Sulfuriferula | 0.04 | -0.20 | -1.94 |
| Bacteria;Proteobacteria;Gammaproteobacteria;Pseudomonadales;Moraxellaceae;Acinetobacter | 0.04 | 0.22 | 1.94 |
| Bacteria;Proteobacteria;Gammaproteobacteria;Xanthomonadales;Rhodanobacteraceae;Dokdonella | 0.02 | 0.21 | 1.88 |
| Bacteria;Proteobacteria;Gammaproteobacteria;Xanthomonadales;Xanthomonadaceae;Luteimonas | 0.04 | 0.17 | 1.74 |

**Table S5.** Correlation between physicochemical parameters and relative abundance of Acidobacteria

|  | Al_2_O_3_ | K_2_O | pH | P_2_O_5_ | Moisture (%) | MgO | Fe_2_O_3_ | Na_2_O | C [%] | TiO_2_ | S [%] | CaO |
| --- | --- | --- | --- | --- | --- | --- | --- | --- | --- | --- | --- | --- |
| Correlation | -0.58 | -0.49 | 0.42 | -0.41 | 0.29 | 0.28 | 0.26 | -0.24 | 0.24 | 0.18 | 0.17 | 0.16 |
| p-value | 0.00 | 0.01 | 0.04 | 0.04 | 0.16 | 0.17 | 0.21 | 0.24 | 0.25 | 0.40 | 0.41 | 0.46 |

**Table S5.** Continued

|  | TOC(ppm) | LOI | MnO | U | H [%] | SiO2 | N [%] |
| --- | --- | --- | --- | --- | --- | --- | --- |
| Correlation | 0.15 | 0.14 | 0.12 | -0.08 | 0.06 | -0.01 | 0.00 |
| p-value | 0.49 | 0.51 | 0.58 | 0.72 | 0.76 | 0.96 | 0.99 |

**
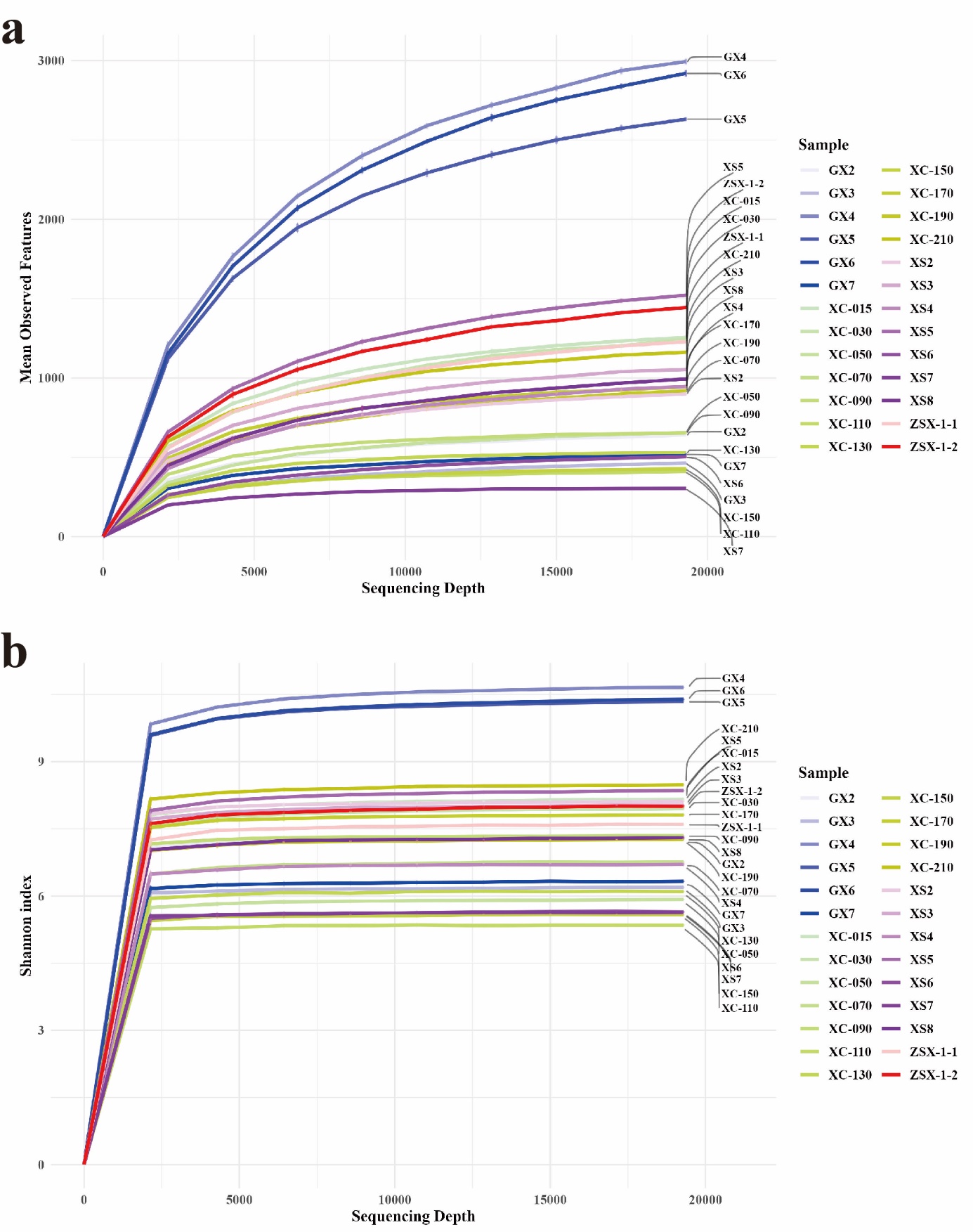
**

**Figure S1.** Rarefaction curves of samples. The x-axis represents sequencing depth with a maximum depth of 19285, which is the minimum reads count after filtering. The y-axis indicates the alpha diversity metrics, a) mean observed features, b) Shannon index for each sample. The curve demonstrated that most samples approached saturation at a sequencing depth of 19285, supporting its suitability as the rarefaction depth for downstream analyses.


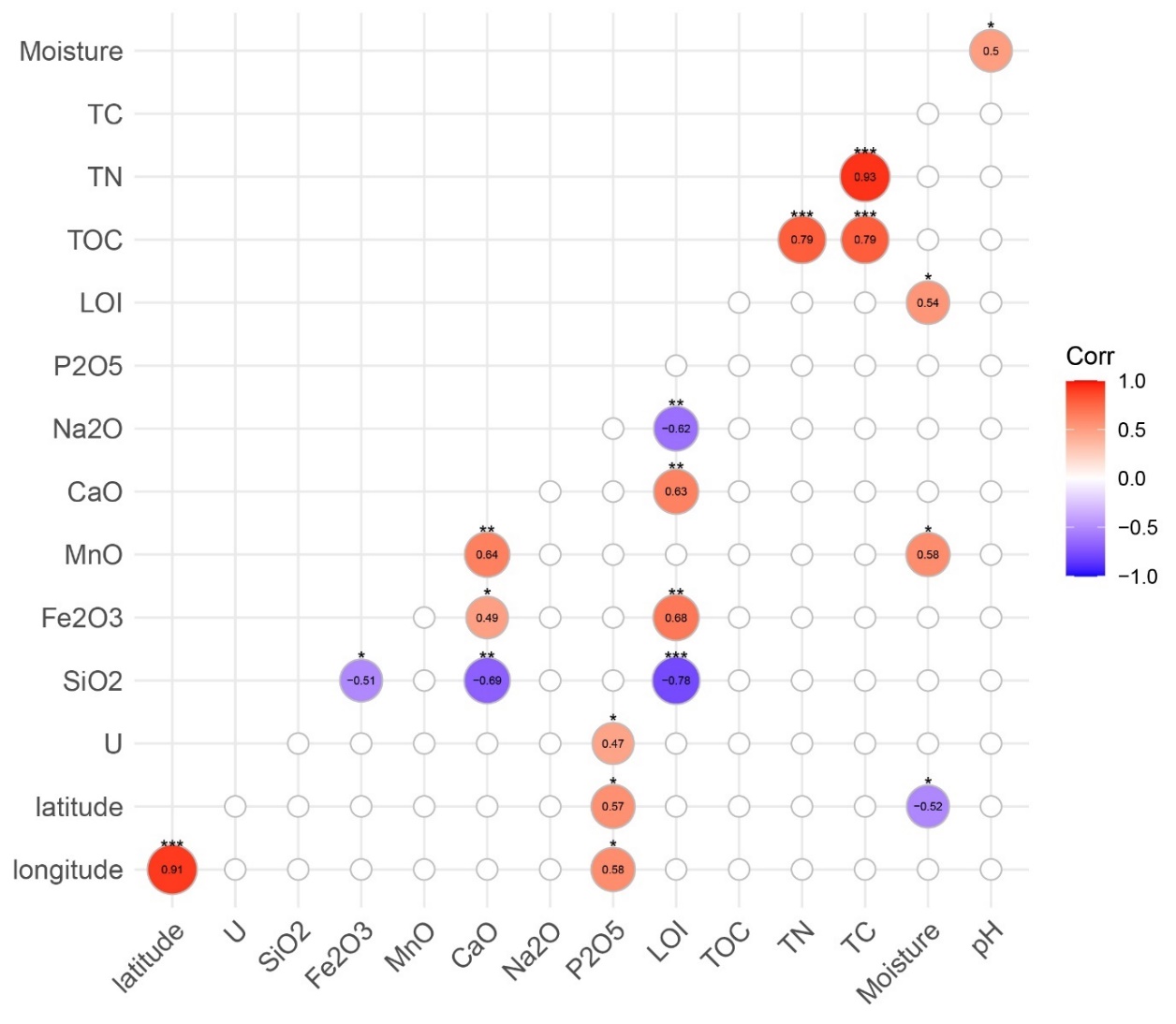


**Figure S2.** Correlation matrix of environmental variables. Red circles denote positive correlations, while blue circles denote negative correlations, with both the color intensity and circle size reflecting the positive or negative correlation and the magnitude of the correlation coefficient (Corr), respectively. Significant correlations were marked with asterisks (*P ≤ 0.05, **P ≤ 0.01, ***P ≤ 0.001). p-values greater than 0.05 are not shown. Abbreviations: TC, Total Carbon; TN, Total Nitrogen; TOC, Total Organic Carbon; LOI, Loss on Ignition.


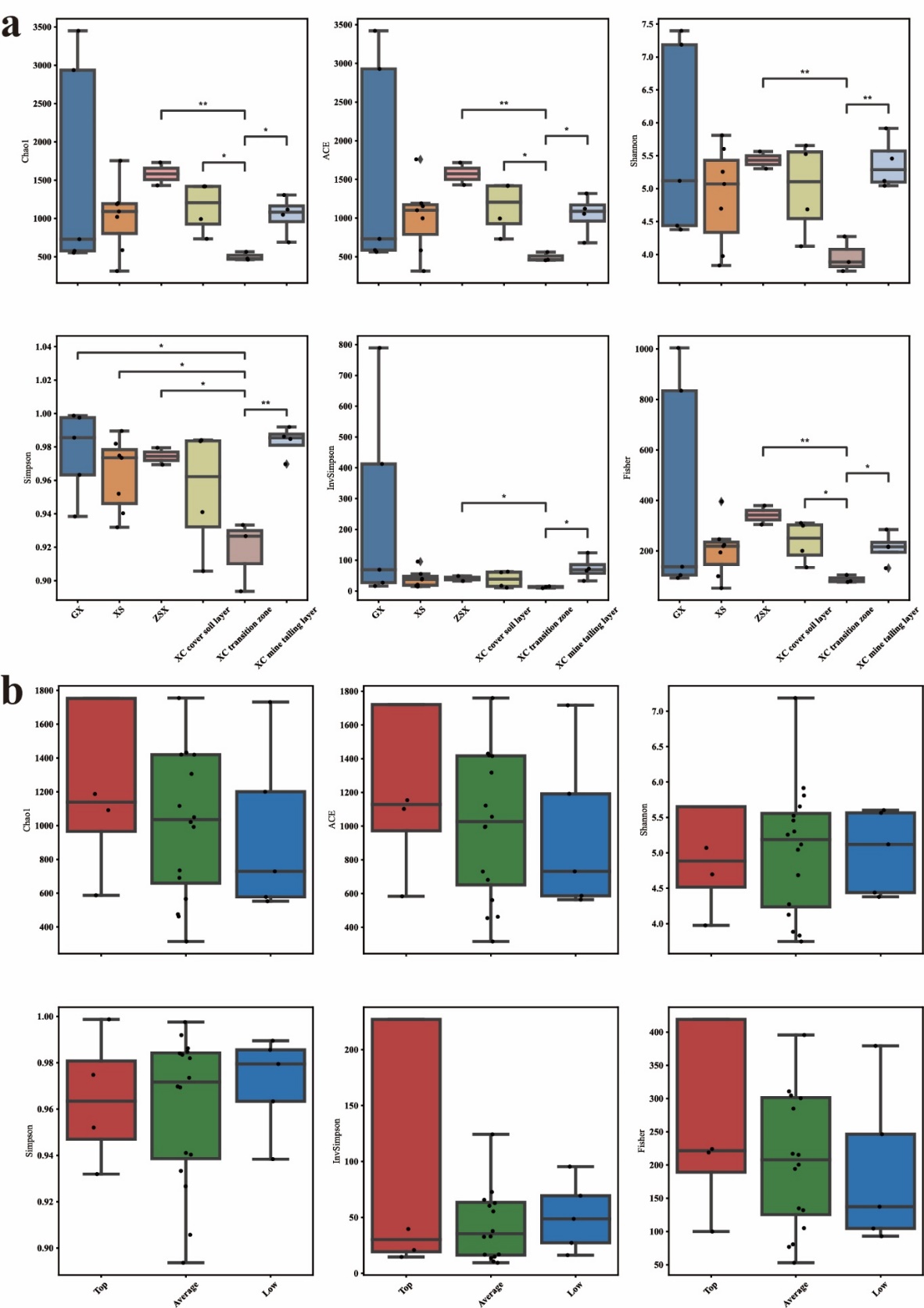


**Figure S3.** Alpha-diversity of microbial communities across different categories. (a) Boxplots of alpha diversity indices (Chao1, ACE, Shannon, Simpson, InvSimpson, and Fisher) for samples from various sites (GX, XS, ZSX) and layers within site XC, including the cover soil layer, transition zone, and mine tailing layer. Statistically significant differences between groups are indicated by asterisks (*P ≤ 0.05, **P ≤ 0.01, ***P ≤ 0.001). (b) Boxplots of the same alpha diversity indices categorized by uranium contamination levels (high, average, and low), with no significant differences observed.
